# Fas/CD95 prevents autoimmunity independently of lipid raft localization and efficient apoptosis induction

**DOI:** 10.1038/ncomms13895

**Published:** 2016-12-23

**Authors:** Anthony C. Cruz, Madhu Ramaswamy, Claudia Ouyang, Christopher A. Klebanoff, Prabuddha Sengupta, Tori N. Yamamoto, Françoise Meylan, Stacy K. Thomas, Nathan Richoz, Robert Eil, Susan Price, Rafael Casellas, V. Koneti Rao, Jennifer Lippincott-Schwartz, Nicholas P. Restifo, Richard M. Siegel

**Affiliations:** 1 Immunoregulation Section, Autoimmunity Branch, National Institute of Arthritis and Musculoskeletal and Skin Diseases (NIAMS), Intramural Research Program, National Institutes of Health (NIH), Bethesda, Maryland 20892, USA; 2 Center for Cell Engineering and Department of Medicine, Memorial Sloan Kettering Cancer Center, New York, New York 10065, USA; 3 Center For Cancer Research, National Cancer Institute (NCI), NIH, Bethesda, Maryland 20892, USA; 4 Cell Biology and Metabolism Program, Eunice Kennedy Shriver National Institute of Child Health and Human Development (NICHD), NIH, Bethesda, Maryland 20892, USA; 5 Immunology Graduate Group, University of Pennsylvania, Philadelphia, Pennsylvania 19104, USA; 6 Clinical Genomics Unit, National Institute of Allergy and Infectious Diseases (NIAID), NIH, Bethesda, Maryland 20892, USA; 7 Genomics and Immunity Branch, NIAMS, Bethesda, Maryland 20892, USA; 8 Center for Cell-Based Therapy, NCI, NIH, Bethesda, Maryland 20892, USA

## Abstract

Mutations affecting the apoptosis-inducing function of the Fas/CD95 TNF-family receptor result in autoimmune and lymphoproliferative disease. However, Fas can also costimulate T-cell activation and promote tumour cell growth and metastasis. Palmitoylation at a membrane proximal cysteine residue enables Fas to localize to lipid raft microdomains and induce apoptosis in cell lines. Here, we show that a palmitoylation-defective Fas C194V mutant is defective in inducing apoptosis in primary mouse T cells, B cells and dendritic cells, while retaining the ability to enhance naive T-cell differentiation. Despite inability to efficiently induce cell death, the Fas C194V receptor prevents the lymphoaccumulation and autoimmunity that develops in Fas-deficient mice. These findings indicate that induction of apoptosis through Fas is dependent on receptor palmitoylation in primary immune cells, and Fas may prevent autoimmunity by mechanisms other than inducing apoptosis.

## Abstract (web summary)

Fas drives apoptosis and mutations in this receptor can cause autoimmunity through failure of cell death. Here, the authors use *lpr/lpr* mice with palmitoylation-defective mutant Fas to provide evidence that Fas might limit spontaneous autoimmunity through a non-apoptotic mechanism.

The TNF-family receptor Fas (CD95, TNFRSF6) directly induces apoptosis in primary cells through recruitment of a death-inducing signalling complex (DISC) consisting of the adapter protein Fas-associated protein with death domain (FADD), the caspase regulator c-FLIP and the death-inducing protease caspase-8. The DISC processes caspase-8 into its active form, which dissociates from the membrane and initiates the proteolytic cascade of apoptosis. Fas triggers cell death in activated T cells, and is critical for restimulation-induced cell death (RICD) through the T-cell receptor (TCR) on CD4^+^ T cells^1^,^2^.

Mutations in the gene encoding the TNF-family receptor Fas (CD95, TNFRSF6) have been linked to autoimmunity for more than 20 years through the discovery of dominant-negative mutations in Fas in autoimmune lymphoproliferative syndrome (ALPS, *MIM 601859*) and a retrotransposon insertion disrupting Fas expression in the *lpr* mouse strain^3^,^4^,^5^,^6^. Both ALPS patients and *lpr* mice spontaneously produce autoantibodies and develop lymphadenopathy and splenomegaly with accumulation of an unusual subset of CD4^−^CD8^−^B220^+^ ‘Double-Negative' (DN) T cells. Increased proportions of CD44^hi^ activated conventional CD4^+^ and CD8^+^ T cells, class-switched B cells and dendritic cells (DCs) also accumulate in Fas-deficient mice^7^,^8^,^9^. Conditional deletion of Fas in T cells, B cells and DC subsets has shown that the accumulation of CD4^−^CD8^−^B220^+^ T cells and DCs is dependent on the cell-intrinsic lack of Fas in T cells and DC subsets, respectively^10^, whereas accumulation of effector T cells is a consequence of autoimmunity and does not depend on lack of Fas on T cells^8^,^10^.

Production of autoantibodies in *lpr/lpr* mice is associated with T-cell help for autoreactive B cells by a population of extrafollicular helper T cells (T_EFH_) expressing the chemokine receptor CXCR4 (ref. 9). The T-cell costimulatory molecule ICOS and ICOS-L, expressed on DCs, are important for accumulation of these T_EFH_ (refs 9, 11). IL-21 and CD40L expressed by these helper T cells are important cytokine signals that promote B-cell class switching and development of autoantibody-secreting B cells in the setting of Fas deficiency^12^,^13^,^14^,^15^,^16^. *Lpr/lpr* mice are a murine model for lupus, as on the B6 background, these mice have increased levels of inflammatory cytokines in the serum, and develop lymphoid infiltrates in multiple organs and glomerular immune complex deposition. On the MRL background, Fas deficiency leads to fatal glomerulonephritis^17^,^18^,^19^.

Defective cell death induced by Fas/CD95 has been assumed to be the underlying cause of escape from immunological self-tolerance in mice and humans with Fas deficiency or dominant-negative mutations. Indeed, defective Fas-induced and TCR-induced death can occur *ex vivo* in T cells from *lpr* mice and ALPS patients. However, the circumstances under which Fas induces cell death are highly restricted. Fas is dispensable for T-cell death after administration of antigens or in the setting of acute infection, with excess pathogen-specific Fas-deficient T cells accumulating only during persistent viral infection^10^,^20^,^21^. Antibody responses to immunogens are normal in Fas-deficient *lpr* mice, but T-cell-independent apoptosis of marginal zone B cells has been demonstrated to depend on Fas–FasL interactions^22^. Development of autoreactive plasma cells is controlled by Fas, although it is not clear that this phenomenon is due to apoptosis *in vivo*^7^,^23^,^24^.

Although Fas is constitutively expressed on the surface of most activated T cells, only effector memory T cells are highly sensitive to Fas-induced cell death^25^. Fas can also perform multiple non-apoptotic functions^26^, including co-stimulation of T-cell activation^27^ and promotion of neuronal differentiation^28^, and tumour cell growth and metastasis^29^,^30^,^31^,^32^. Distinguishing the apoptotic from non-apoptotic functions of Fas is therefore critical to understanding its functions *in vivo*.

Receptor and ligand clustering into higher-order structures is required for efficient apoptotic signalling through Fas. Oligomerization of caspase-8 is critical for activation of its enzymatic activity^33^ and the death effector domain (DED) of FADD and caspase-8 can oligomerize independently of the receptor^34^,^35^. Quantitative studies of the signalling complexes initiated by Fas and related receptors for the TNF-family cytokine TRAIL have shown that caspase-8 is present in higher amounts than the adapter protein FADD, leading to a model in which caspase-8 forms self-aggregated chains leading to efficient activation of the apoptotic caspase cascade^36^,^37^. Variability in the concentrations of apoptotic regulators can also influence the susceptibility of individual cells to death receptor-induced apoptosis^38^, perhaps by regulating the efficiency of assembly of complexes that activate caspase-8.

Partitioning of Fas into lipid raft microdomains promotes apoptosis signalling^39^,^40^. Palmitoylation of Fas at a cysteine just internal to the plasma membrane (C194 in mouse and C199 in human) is critical for directing lipid raft localization of the receptor, efficient assembly of the Fas-FADD-caspase-8 DISC, receptor internalization and apoptosis induction in transfected cell lines^41^,^42^. However, the extent to which Fas palmitoylation and lipid raft localization controls Fas-mediated apoptosis of primary immune cells and contributes to the phenotype of Fas deficiency is not known. To address this question, we generated mice in which the palmitoylation site of Fas responsible for lipid raft localization is mutated (FasC194V^*lpr/lpr*^). Palmitoylation-deficient Fas was severely defective in mediating FasL-induced apoptosis in primary B, T and DCs, as well as restimulation-induced cell death in CD4^+^ T cells. However, this Fas mutant could still promote T-cell differentiation, a recently recognized non-apoptotic function of the receptor. FasC194V^*lpr/lpr*^ mice were also protected from all the autoimmune features of Fas deficiency on an *lpr/lpr* background. These results confirm the importance of Fas palmitoylation and lipid raft localization for efficient induction of apoptosis and suggest that non-apoptotic functions of Fas may be more important for protection from autoimmunity than has been previously appreciated.

## Results

### Palmitoylation of Fas promotes receptor microclustering

**Figure 1:**
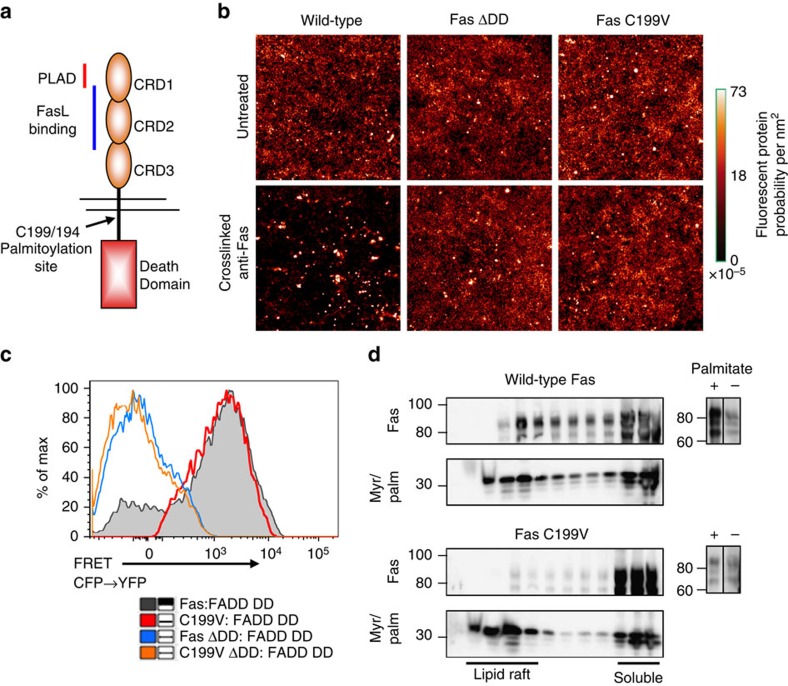
Characterization of palmitoylation-mutant Fas. (**a**) Schematic of the Fas receptor, showing the location of palmitoylation site Cys199 (C194 in mouse). CRD: Cysteine-rich domain. PLAD: Pre-ligand assembly domain. (**b**) COS-7 cells were transfected with mEos2-tagged full-length WT, C199V Fas or WT Fas receptor lacking the death domain (ΔDD) and treated with crosslinked anti-Fas antibody. Individual cells were analysed via super-resolution PALM imaging. Minimum three cells were imaged (*N*=3–6) by PALM, with data representative from a single cell per treatment. Composite images were generated by combining single molecules localized in a PALM time series (3,000–5,000 images total). (**c**) HEK293T cells transfected with a truncated FADD (death domain only; FADD DD) and WT or C199V Fas receptor, either full-length or lacking the death domain (ΔDD), were analysed for interactions by FRET-based flow cytometry. Results are representative of two independent experiments. (**d**) HEK293T cells were transfected with a plasmid coding for a cyan-fluorescent protein (CFP) modified by signals for myristoylation and palmitoylation (MyrPalm), which targets CFP to lipid rafts^65^, as well as constructs containing WT or palmitoylation-deficient Fas C199V. Immunoblot analysis for Fas distribution was performed on Optiprep step gradient fractions of cell lysates 48 h post-transfection. WT or mutant C199V Fas-transfected HEK293T cells were incubated in the presence or absence of azide-linked palmitic acid and lysates were subjected to biotin-amide click chemistry. Immunoprecipitation of lysates was performed using streptavidin agarose beads and analysed for Fas by immunoblotting. Data are representative of three independent experiments.

On binding to Fas ligand, Fas clusters into microscopically visible structures that depend on an intact Fas death domain (DD), and which is required for effective apoptosis signalling^39^. To determine whether palmitoylation affected receptor clustering after ligation, we imaged membrane-localized Fas with super-resolution photoactivated localization microscopy (PALM) (Fig. 1a). At steady-state, all forms of Fas had a random distribution across the plasma membrane (Fig. 1b, upper panels). After anti-Fas mAb stimulation, full-length wild-type (WT) Fas formed discrete non-random clusters of receptors, while a Fas DD-deleted mutant that cannot bind FADD or signal effectively for cell death (Fas ΔDD), failed to form receptor clusters, similar to what was observed previously with conventional confocal microscopy^39^. Strikingly, the C199V Fas palmitoylation mutant was as defective at forming receptor clusters as the death domain mutant (Fig. 1b, lower panels).

Since previously described Fas mutants that fail to cluster after receptor crosslinking cannot recruit FADD^39^, we next asked whether the Fas C199V mutant has an intrinsic defect in binding to FADD. We used fluorescence resonance energy transfer (FRET) between Fas and FADD proteins fused to cyan fluorescent protein (CFP) and yellow fluorescent protein (YFP), respectively, to measure these interactions in living cells. These experiments showed that the C199V Fas mutant interacted with FADD as well as WT Fas, while the truncated ΔDD variants were unable to interact with FADD (Fig. 1c). Density gradient fractionation of transfected cell lysates confirmed that the Fas C199V protein, unlike WT Fas, could not localize to lipid rafts, and palmitate labelling using click chemistry showed that the palmitoylation of the Fas C199V protein was reduced relative to the WT Fas control (Fig. 1d). Thus, the primary function of Fas lipid raft localization appears to be to support higher-order clustering of the receptor rather than the ability to recruit FADD.

### Fas C194V mutant impairs apoptosis in primary T cells

**Figure 2:**
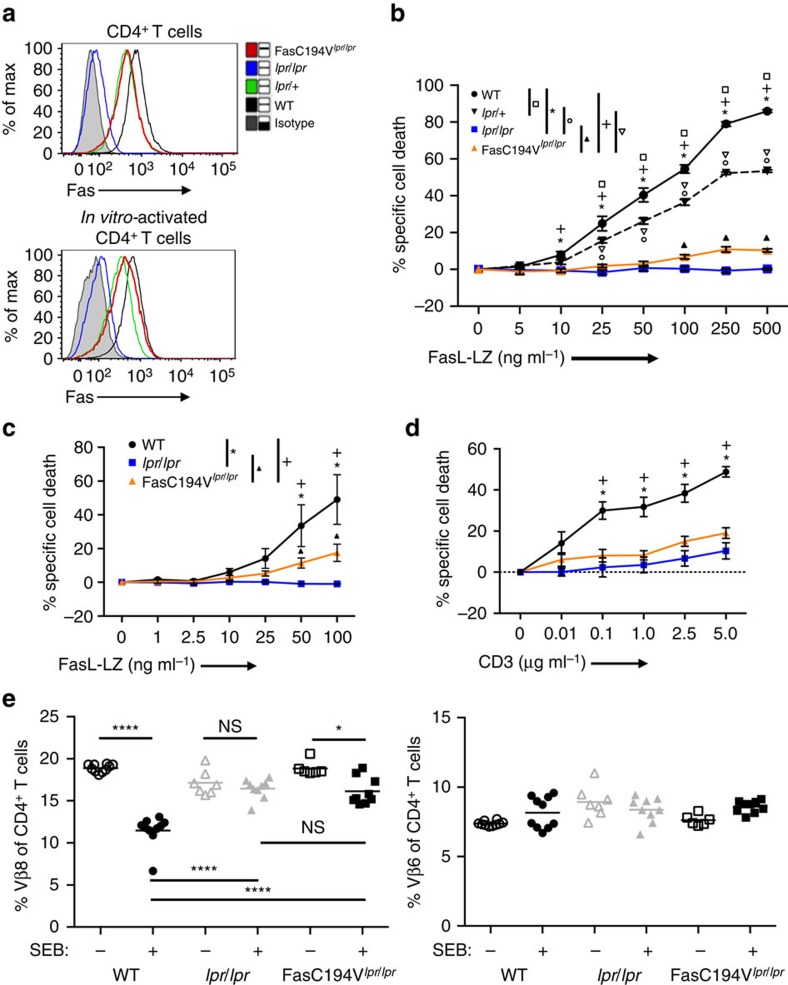
Fas C194V is defective in inducing T-cell apoptosis. (**a**) Flow cytometry was performed on *ex vivo* resting and *in vitro*-activated and cultured CD4^+^ T cells of WT, *lpr*/+, *lpr/lpr* or FasC194V^*lpr/lpr*^ mice. Grey shaded: isotype control; Black: WT; Green: *lpr*/+; Blue: *lpr/lpr*; Red Bold: FasC194V^*lpr/lpr*^. (**b**) *In vitro*-activated CD4^+^ T cells from WT, *lpr/*+, *lpr/lpr* or FasC194V^*lpr/lpr*^ mice were cultured in the presence of increasing amounts of FasL-LZ for 8 h before analysis for apoptosis by flow cytometry. Data are cumulative of six independent experiments and represents the mean specific cell death±s.e.m. (**c**) Apoptosis of gated effector CD4^+^ T cells (TCRβ^+^B220^−^CD4^+^CD44^hi^CD62L^lo^) within lymphocytes from the spleen and lymph nodes of WT, *lpr/lpr*, or FasC194V^*lpr/lpr*^ mice were stimulated with FasL-LZ and analysed as in **b** (*N*=6). (**d**) *In vitro*-activated purified CD4^+^ T cells from the indicated genotypes were stimulated with plate-bound anti-CD3 for 8 h before apoptosis analysis as in (**b**). Results are compiled from six independent experiments (*N*=6) and represent the mean specific cells death ± s.e.m. (**e**) CD4^+^Vβ8^+^ T cells were analysed in 8–10-week-old WT, *lpr/lpr*, or FasC194V^*lpr/lpr*^ mice 6 days after four daily injections of either PBS or 20 μg SEB IP. Data are representative of two experiments, with *N*≥6 for each genotype. No statistically significant reductions in the percentages of Vβ6^+^CD4^+^ T cells were seen in any genotype. Legend for statistical significance in (**b**–**d**): *P≤0.001, WT versus *lpr/lpr*.^+^*P*≤0.001, WT versus FasC194V^*lpr/lpr*^. Square: *P*≤0.001, WT versus *lpr*/+. Circle: *P*≤0.001 *lpr*/+ versus *lpr/lpr*. Inverted triangle: *P*≤0.001, *lpr/*+ versus FasC194V^*lpr/lpr*^. Filled triangle: * P*≤0.001 *lpr/lpr* versus FasC194V^*lpr/lpr*^. Unpaired *t*-test was used for statistical calculations. For (**e**), NS=not significant, *P<0.05, ****P<0.0001 by Mann–Whitney.

To investigate the function of Fas lipid raft localization in primary immune cells *in vivo*, we generated transgenic mice with a bacterial artificial chromosome (BAC) encoding the Fas protein engineered to express a Fas C194V mutant, the mouse equivalent of the Fas C199V mutation (Supplementary Fig. 1a). The founder line expressing the highest level of the Fas C194V transgene was backcrossed onto the C57BL/6 *lpr/lpr* Fas-deficient strain, and subsequently interbred to generate homozygous transgenic mice that maximally express Fas from the BAC transgene and negligible amounts of WT Fas. These mice will henceforth be referred to as FasC194V^*lpr/lpr*^. Levels of surface Fas in FasC194V^*lpr/lpr*^ mice were intermediate between that on *lpr/lpr* and WT mice, and were more comparable to heterozygous *lpr*/+ mice (Fig. 2a; Supplementary Fig. 1b). Western blot analyses of cell lysates from activated T cells showed Fas protein expression in FasC194V^*lpr/lpr*^ to be comparable to that found in *lpr*/+ mice (Supplementary Fig. 1c).

Fas can directly induce apoptosis in activated T cells, and participates in the apoptosis of CD4^+^ T cells restimulated through the T-cell receptor^43^,^44^,^45^. To assess the death-inducing function of the Fas C194V transgenic receptor, we treated *in vitro-*activated CD4^+^ T cells with a form of soluble FasL oligomerized through an isoleucine zipper motif (FasL-LZ) that potently induces T-cell death in WT cells^25^. FasL-induced apoptosis in activated FasC194V^*lpr/lpr*^ T cells was reduced to levels only slightly above that of Fas-deficient *lpr/lpr* T cells (Fig. 2b). Importantly, *lpr/+* T cells with cell surface levels of comparable to that found on FasC194V^*lpr/lpr*^ mice were much more susceptible to Fas-induced apoptosis than FasC194V^*lpr/lpr*^ T cells, with levels of cell death nearly as high as that in WT T cells (Fig. 2b). These data show that the defect in Fas-induced apoptosis exhibited by FasC194V^*lpr/lpr*^ T cells cannot be attributed to reduced receptor surface expression. Effector memory CD4^+^ T cells are sensitive to Fas-induced apoptosis even in the absence of prior T-cell activation^25^, and *ex vivo* FasC194V^*lpr/lpr*^ CD4^+^ effector T cells had reduced sensitivity to Fas-induced apoptosis, with a small amount of residual cell death (Fig. 2c). When activated CD4^+^ T cells were restimulated through the TCR, FasC194V^*lpr/lpr*^ cells were highly resistant to TCR-induced apoptosis, with levels of cell death comparable to *lpr/lpr* T cells (Fig. 2d). To determine if the Fas C194V mutant could mediate T-cell deletion *in vivo*, we treated mice with Staphylococcal Enterotoxin B (SEB), which is known to delete CD4^+^ Vβ8 expressing T cells in a Fas-dependent manner after repeated administration^46^,^47^. Consistent with previously reported findings, *lpr/lpr* mice were resistant to SEB-induced deletion of Vβ8^+^CD4^+^ T cells. In FasC194V^*lpr/lpr*^ mice, Vβ8^+^ cell deletion was significantly impaired, and the remaining percentage of Vβ8^+^CD4^+^ T cells after SEB treatment was no different than in *lpr* mice (Fig. 2e). Collectively, these data show that palmitoylation and lipid raft localization are necessary for Fas to induce apoptosis in primary T cells, and further demonstrate that the Fas C194V mutant is deficient in rescuing the apoptotic defects in Fas-deficient autoimmune prone *lpr/lpr* mice *in vitro* and *in vivo.*

### Fas C194V mutant enhances T-cell differentiation

**Figure 3:**
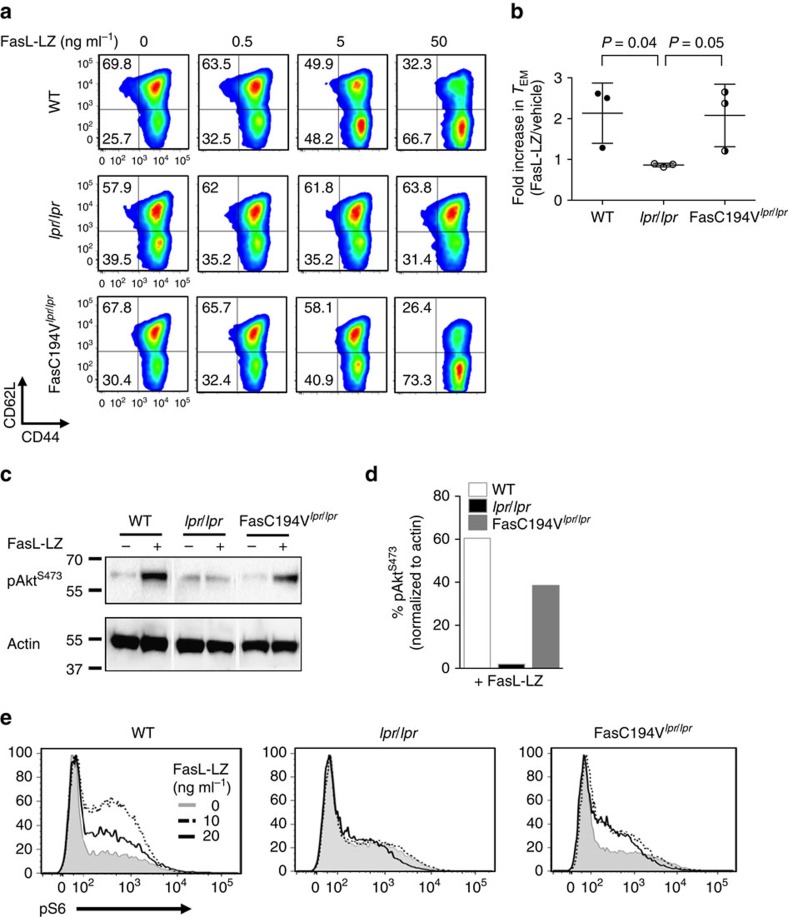
The Fas C194V mutant mediates precocious T-cell differentiation. (**a**) Naive CD4^+^ T cells from 10-week-old WT, *lpr/lpr* or FasC194V^*lpr/lpr*^ mice were stimulated for 48 h with anti-CD3/CD28 in the presence or absence of the indicated doses of FasL-LZ. Activated cells were subsequently expanded for 96 h in media with IL-2 in the presence or absence of FasL and subjected to flow cytometry after staining for CD44 and CD62L to delineate central and effector memory cell subsets. Fold increase of effector memory (T_EM_) in cells treated with FasL-LZ versus control is summarized in **b**, with *P*=0.04 for WT versus *lpr/lpr* and *P*=0.05 for FasC194V^*lpr/lpr*^versus *lpr/lpr*. There was no significant difference between WT and FasC194V^*lpr/lpr*^samples. Unpaired *t*-test was used to calculate statistical significance. Data are representative of three independent experiments (*N*=3) representing mean ± s.e.m. (**c**) Equal numbers of 6 day-activated naive cells, stimulated in the presence or absence of 10 ng ml^−1^ FasL-LZ, from the indicated genotypes of mice were lysed and immunoblotted for pAkt^S473^ and actin (loading control). (**d**) pAkt^S473^ was quantitated from the blots in **c** by densitometry of pAkt^S473^ levels normalized to actin. The results are representative of two independent experiments (*N*=2). (**e**) Intracellular staining for pS6 was performed on CD4^+^ T cells activated as in **a** for 6 days with FasL at the indicated doses. Data are representative of two independent experiments (*N*=2).

To determine whether the Fas C194V mutant could still signal for other functions besides cell death, we examined the ability of Fas to trigger enhanced T-cell differentiation. We have recently shown that Fas can act in concert with the TCR to drive naive CD8^+^ T cells towards a CD44^hi^CD62L^lo^ cytotoxic effector phenotype, a process termed precocious differentiation^48^. Enhanced T-cell differentiation was mediated by FasL expressed on memory T cells and resulted in reduced survival of tumour-specific T cells and impaired antitumor efficacy in adoptive T-cell transfer experiments^48^. To determine if Fas can mediate precocious T-cell differentiation of CD4^+^ T cells and test the ability of the Fas C194V mutant to mediate this non-apoptotic function of Fas, naive CD4^+^ T cells were stimulated *ex vivo* with anti-CD3/28 in concert with FasL-LZ. Provision of FasL-LZ enhanced the differentiation of naive CD4^+^ cells into CD44^hi^CD62L^lo^ effector memory cells in cultures of both WT and FasC194V^*lpr/lpr*^ T cells, whereas *lpr/lpr* T cells were completely refractory to enhancement of effector memory T-cell differentiation by FasL-LZ (Fig. 3a,b).

In addition to changes in surface phenotype, we also sought biochemical evidence that the Fas C194V mutant could activate non-apoptotic signalling pathways in CD4^+^ T cells. Because Fas signalling has been linked to activation of the Akt pro-survival signalling pathway in non-immune cells^28^,^32^, we asked whether the Akt pathway was also activated during precocious differentiation of CD4^+^ T cells. Co-stimulation by FasL-LZ increased phosphorylation of Akt at position 473, (pAkt^S473^) in both WT and to a lesser extent in FasC194V^*lpr/lpr*^, but not *lpr/lpr* T-cell blasts (Fig. 3c,d). Similar results were seen with phosphorylation of the ribosomal S6 protein, an important anabolic target of Akt and mTOR, by both intracellular flow cytometry and western blot analysis (Fig. 3e; Supplementary Fig. 2). Taken together, these data show that although the Fas C194V mutant cannot efficiently induce cell death, it still functions to promote effector memory T-cell differentiation and enhance activation of the Akt signalling pathway.

### Fas C194V prevents the Fas deficiency phenotype

**Figure 4:**
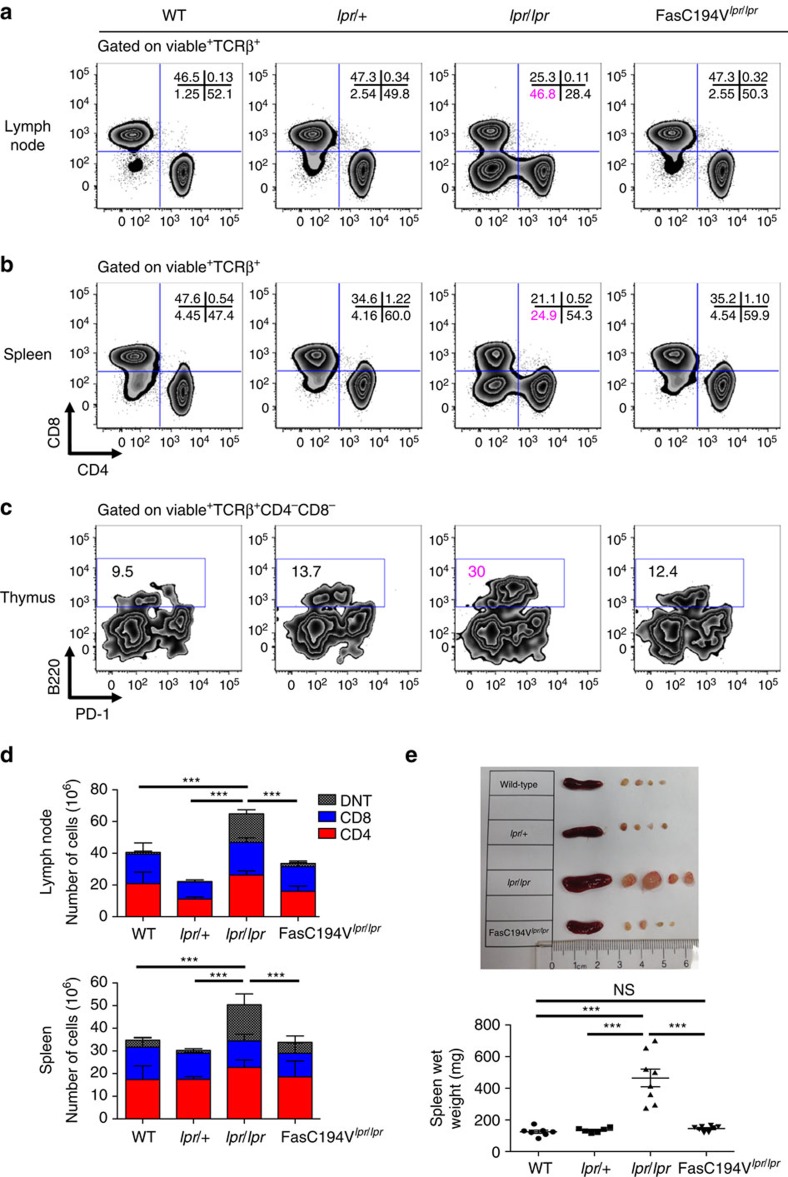
Fas C194V prevents lymphoaccumulation associated with Fas deficiency. Lymph nodes (**a**), spleens (**b**) and thymi (**c**) of age-matched WT, *lpr/*+, *lpr/lpr* and FasC194V^*lpr/lpr*^ mice were isolated, stained for the indicated surface markers and analysed by flow cytometry, with quantitation and statistical analysis (unpaired *t*-test) of DN T cells in **d**. Data are compiled from three independent experiments, represented as mean ± s.e.m. ***P≤0.001. Viable cells were gated TCRβ^+^ for both lymph nodes (**a**) and spleen (**b**) before analysis. Thymic cells (**c**) were gated TCRβ^+^CD4^−^CD8^−^ on the viable population before analysis. Mice were ∼30 weeks of age (*n*≥12 for each genotype). (**e**) Representative lymph nodes and spleens of the indicated age-matched genotypes, with spleen wet weights. Image representative of two independent experiments. Mice were ∼30 weeks of age. Quantitation is cumulative of three independent experiments±s.e.m. Mann–Whitney test was used for statistical analysis. ***P≤0.001. NS=not significant.

A central characteristic shared by ALPS patients and *lpr/lpr* Fas-deficient mice is the development of lymphoproliferative disease, characterized by splenomegaly, lymphadenopathy and accumulation of CD4^−^CD8^−^ DN T cells^3^,^4^. To determine whether lipid raft localization of Fas is necessary to prevent the immunological consequences of Fas deficiency, we examined the peripheral lymphoid tissues of FasC194V^*lpr/lpr*^ mice. Strikingly, despite being unable to mediate efficient apoptosis, the presence of the Fas C194V mutant receptor completely prevented the accumulation of DN T cells in the lymph nodes and spleen characteristic of Fas-deficient *lpr/lpr* mice at up to 30 weeks of age (Fig. 4a,b). These findings were confirmed using the B220 CD45 isoform, which is expressed on DN T cells, which accumulated in the spleens of *lpr/lpr*, but not FasC194V^*lpr/lpr*^ mice even after 6 months of age (Supplementary Fig. 3a). B220 was expressed on *lpr/lpr* DN T cells, with minimal expression observed on the small population of DN T cells present in WT, *lpr*/+ or FasC194V^*lpr/lpr*^ cells, (Supplementary Fig. 3b). To further characterize and quantify the DN T-cell population over time, the percentage of splenic and lymph node B220^+^TCRβ^+^ T cells were analysed every 6 weeks starting from 12 to 30 weeks of age (Supplementary Fig. 3b). *Lpr/lpr* mice had significantly higher percentages of B220^+^TCRβ^+^ cells at all time points assessed, whereas these cells did not accumulate in FasC194V^*lpr/lpr*^ or WT mice. The thymus contains a small population of TCRαβ-expressing CD4^−^CD8^−^ T cells that co-express PD-1 and survive negative selection to become the precursors of CD8αα-expressing intestinal epithelial lymphocytes^49^. In *lpr/lpr* mice, recirculating CD4^−^CD8^−^B220^+^ T cells can be detected in the thymus, and the development of PD-1^+^ DN T cells is not affected (Fig. 4c). The Fas C194V transgene reduced the frequency of thymic CD4^−^CD8^−^B220^+^ T cells to levels similar to that of WT mice, even in 30-week-old-aged mice (Fig. 4c). Histological analysis of the lung and liver demonstrated that the Fas C194V transgene largely prevented the lymphocytic infiltrates that accumulate in these tissues of Fas-deficient *lpr/lpr* mice (Supplementary Fig. 4).

Inspection of lymph nodes and spleen and quantitation of CD4^+^, CD8^+^ and CD4^−^CD8^−^B220^+^ DN T cells showed that the Fas C194V transgene completely protected mice from the lymphadenopathy and splenomegaly that arises in Fas-deficient *lpr/lpr* mice. This was due largely to the prevention of accumulation of DN T cells (Fig. 4d,e; Supplementary Fig. 3). Like Fas C194V mutant mice, *lpr/+* mice do not develop the *lpr* syndrome even at 30 weeks of age. However, unlike the Fas C194V receptor, which is unable to mediate efficient apoptosis in T cells, Fas-induced apoptosis in the *lpr*/+ T cells remained relatively intact (Fig. 2). Studies with tissue-specific deletion of Fas have shown that accumulation of DN T cells is T-cell-intrinsic^10^. Taken together, these results suggest that within the T-cell compartment, non-apoptotic functions of the Fas C194V receptor, rather than its classic apoptosis-inducing function, prevents lymphoaccumulation of DN T cells.

### The Fas C194V mutant receptor prevents autoimmunity

**Figure 5:**
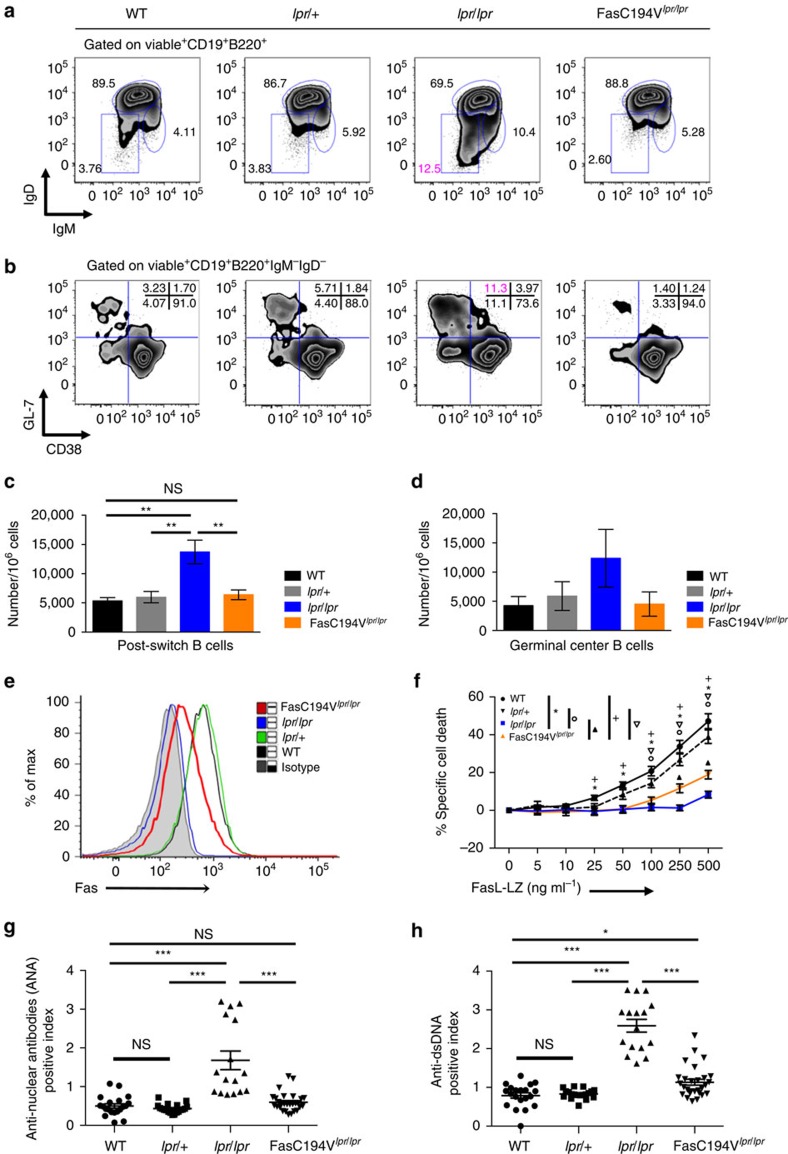
Palmitoylation-deficient Fas prevents autoantibody production. (**a**) Spleens from 30 week-old WT, *lpr/*+, *lpr/lpr* and FasC194V^*lpr/lpr*^ mice were isolated and stained for surface IgM and IgD expression in B cells. All cells are gated TCRβ^−^CD19^+^B220^+^ in the viable cell pool. Results are representative of six independent experiments (*N*=6). (**b**) Fresh splenocytes from the indicated genotypes were stained for CD38 and GL-7. All cells are gated TCRβ^−^CD19^+^B220^+^IgM^−^IgD^−^ in the viable cell pool. (**c**) Quantitation of splenic IgM^−^IgD^−^ post-switch B cells. Results are compilation of six independent experiments (*N*=6), represented as mean ± s.e.m. Mann–Whitney test was used for statistical analysis. **P≤0.01. NS=Not significant. (**d**) Quantitation of CD38^−^GL-7^+^ germinal center B cells. (**e**) Surface expression of Fas in mice of the indicated genotypes was analysed on splenic B cells activated with LPS for 72 h. Grey shaded: isotype control; Black: WT; Green: *lpr*/+; Blue: *lpr/lpr*; Red: FasC194V^*lpr/lpr*^. Data are representative of three independent experiments (*N*=3). (**f**) LPS-activated B cells were incubated with increasing amounts of FasL-LZ for 8 h before analysis for cell death via flow cytometry. Results are compiled from five independent experiments (*N*=5), with data shown as mean ± s.e.m. *P≤0.001, WT versus *lpr/lpr*. ^+^*P*≤0.001, WT versus FasC194V^*lpr/lpr*^. Circle: *P*≤0.001 *lpr*/+ versus *lpr/lpr*. Inverted triangle: *P*≤0.001, *lpr/*+ versus FasC194V^*lpr/lpr*^. Filled triangle: *P*≤0.001 *lpr/lpr* versus FasC194V^*lpr/lpr*^. Unpaired *t*-test was used for statistical calculations. (**g**,**h**) Serum was collected from WT, *lpr*/+, *lpr/lpr* and FasC194V^*lpr/lpr*^ age-matched mice (≥24–36 weeks, or 6–9 months; *N*≥15 for each genotype) and analysed via ELISA for antibodies to nuclear proteins (ANA; (**g**)) and double-stranded DNA (dsDNA; (**h**)). Experiment is representative of three independent (*N*=3). experiments±s.e.m. Mann–Whitney was used for statistical analysis. *P≤0.05. ***P≤0.0001. NS=Not significant.

Increased production of autoantibodies and abnormally activated B cells can be seen in mice lacking Fas expression in B cells^10^,^50^. We therefore investigated whether the Fas C194V mutant is able to prevent spontaneous B-cell activation and autoantibody production. As expected, *lpr/lpr* mice accumulated significantly increased numbers of class-switched (IgM^−^IgD^−^) B cells, and increased germinal center phenotype (CD38^lo^GL-7^hi^) B cells (Fig. 5a–d). The FasC194V^*lpr/lpr*^ mice completely reversed the accumulation of these activated B-cell subsets, with percentages of class-switched B cells at or below those found in WT mice. This was observed in mice up to 30 weeks of age (Fig. 5a–d). Concordantly, elevated levels of IgG, IgM and IgA were observed in the serum of ≥6-month-old *lpr/lpr* mice, but IgG levels in FasC194V^*lpr/lpr*^ were comparable with WT mice, and IgM and IgA were much reduced by the Fas C194V transgene than levels observed in *lpr* mice (Supplementary Fig. 5).

To determine whether the effects of the Fas C194V mutant were due to apoptosis induction in B cells, we activated splenic B cells with LPS and treated them with FasL-LZ. Activated FasC194V^*lpr/lpr*^ B cells expressed surface Fas, but at levels intermediate between *lpr/lpr* and *lpr/+* B cells (Fig. 5e). However, while FasL-LZ treatment induced apoptosis nearly equivalently in *lpr/+* and WT B cells, FasC194V^*lpr/lpr*^ were almost as defective in Fas-induced apoptosis as *lpr/lpr* B cells (Fig. 5f). Taken together, these data show that the Fas C194V mutant, while defective in inducing B-cell death, could prevent the accumulation of post-switch and germinal center B cells and hyper-production of immunoglobulin in the setting of Fas deficiency. This implicates non-apoptotic signalling through Fas in preventing the accumulation of activated B cells and limiting antibody production.

Although they do not develop severe nephritis, Fas-deficient *lpr/lpr* mice 6 months of age or older on the B6 background produce high titres of anti-nuclear (ANA) and anti-double-stranded DNA (dsDNA) antibodies, which can deposit and fix complement in the glomeruli. To determine whether the apoptosis-defective Fas C194V mutant can also abrogate these features of Fas deficiency, we measured ANA and anti-dsDNA antibodies in FasC194V^*lpr/lpr*^mice between 6 and 9 months old. Concomitant with the lack of accumulation of class-switched B cells, age-matched FasC194V^*lpr/lpr*^ mice possessed significantly lower levels of serum anti-nuclear and anti-dsDNA antibodies compared with *lpr* mice (Fig. 5g,h). Histologically visible immune cell infiltrates and albuminuria was minimal in *lpr/lpr* mice with or without the Fas C194V transgene in mice at 8 months of age (Supplementary Fig. 6). However, immunohistochemistry revealed scattered infiltrates of CD3^+^ and F4/80^+^ cells in B6 *lpr* mice that were reduced by the Fas C194V transgene (Supplementary Fig. 7a,b). Immune complex deposition was also reduced in *lpr/lpr* mice harbouring the Fas C194V transgene, with both IgG and complement C3 levels reduced in comparison to *lpr* mice (Supplementary Fig. 7c–f). Thus, despite defective apoptosis-inducing ability in both T and B cells, the Fas C194V mutant could substantially protect aged mice from hypergammaglobulinemia, production of anti-nuclear autoantibodies, and the mild autoimmune sequellae that develop in Fas-deficient mice on the B6 background.

**Figure 6:**
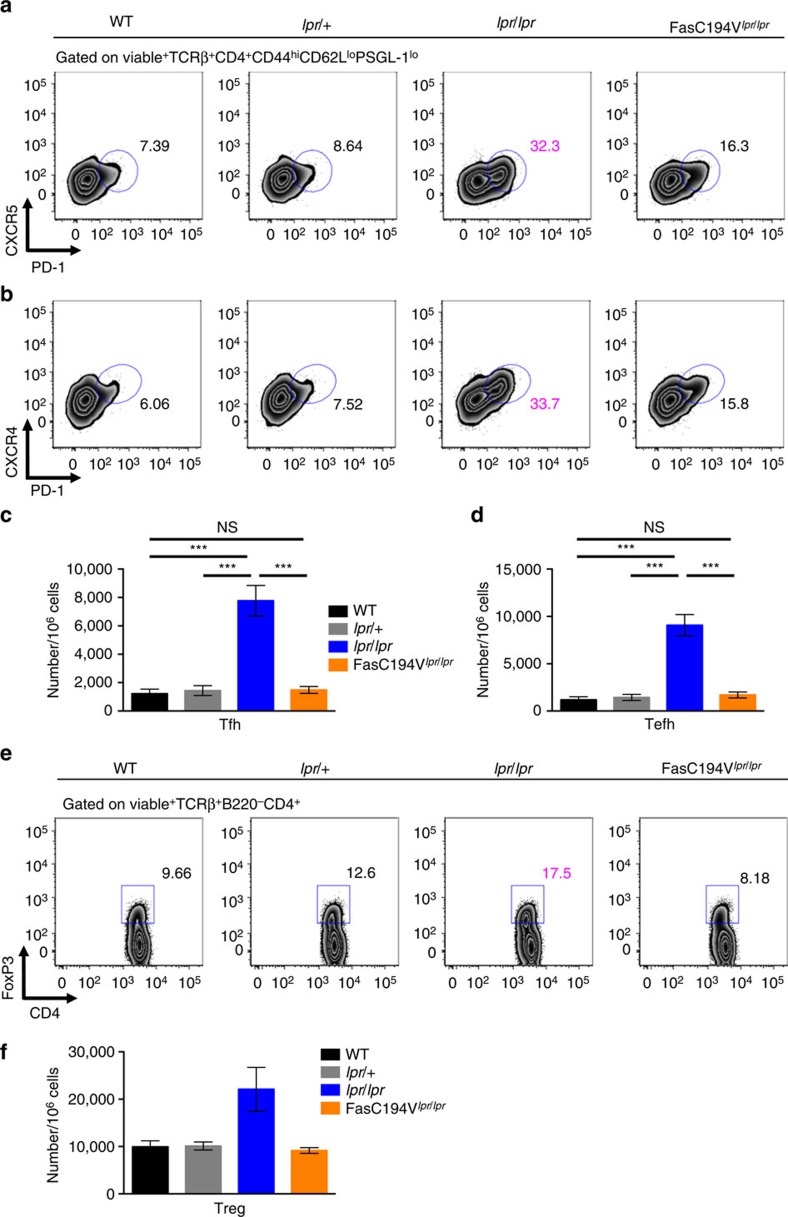
Fas C194V prevents accumulation of T_FH_ and T_EFH_. Spleens from 30-week-old WT, *lpr/*+, *lpr/lpr* and FasC194V^*lpr/lpr*^ mice were isolated and stained for surface markers to CD4^+^ T follicular (T_FH_; (**a**)) and T extrafollicular (T_EFH_; (**b**)) cells and analysed by flow cytometry. All cells were gated TCRβ^+^B220^−^CD4^+^CD44^hi^CD62L^lo^PSGL-1^lo^ in the viable cell pool before analysis. Data are representative of six independent experiments (*N*=6). Quantitation for both splenic T_FH_ (**c**) and T_EFH_ (**d**) performed with statistical analysis using Mann–Whitney test. Data are cumulative of 6 independent experiments (*N*=6)±s.e.m. ***P≤0.001. NS=Not significant. (**e**) Splenocytes from 24-week-old mice of the indicated genotypes were stained for markers to T regulatory cells. All cells were gated TCRβ^+^B220^−^CD4^+^ in the viable cell pool before analysis. Data are representative of three independent experiments (*N*=3), with quantitation performed in **f** showing data as mean ± s.e.m.

Autoantibodies in Fas-deficient animals bear the hallmarks of T-cell help, such as somatic hypermutation and class switching^51^. Reduction of autoantibody production after blockade of critical signals delivered by helper T cells to B cells (such as ICOS, CD40L and IL-21) supports a role for T-cell help in promoting autoimmunity in the setting of Fas deficiency^9^,^12^,^15^. To investigate whether the Fas C194V transgene affects T-cell help associated with Fas deficiency, we examined T helper subsets in FasC194V^*lpr/lpr*^ mice. Activated CD44^hi^ effector T cells accumulate in a B-cell-dependent manner in *lpr* mice lacking Fas^8^. Concordant with the lack of autoantibody production in FasC194V^*lpr/lpr*^ mice, accumulation of CD44^hi^CD62L^lo^ CD4^+^ effector T cells in *lpr/lpr* mice was reduced, but not completely eliminated, by the Fas C194V transgene (Supplementary Fig. 8). In addition, accumulation of both follicular (CXCR5^+^PD-1^hi^) and extrafollicular (CXCR4^+^PD-1^hi^) helper T cells in the spleen was abrogated in aged FasC194V^*lpr/lpr*^ mice (Fig. 6a–d). Concordantly, levels of pro-inflammatory and B-cell stimulatory cytokines IL-6, TNF, IL-17A, IL-17F and IL-21 were elevated in the serum of >6-month-old *lpr/lpr* mice, but reduced in FasC194V^*lpr/lpr*^ to levels similar to WT mice (Supplementary Fig. 9a–e).

Although Fas is not required for the development of regulatory T cells, there is some evidence that Tregs play a role in restraining the development of anti-DNA secreting B cells^52^. It is possible that the Fas C194V transgene may suppress autoimmunity in *lpr* mice by promoting the development of Tregs. While there was some accumulation of FoxP3^+^CD4^+^ Tregs in 6-month-old *lpr* mice, the Fas C194V transgene reduced the frequency of Tregs back to levels comparable to that of WT controls (Fig. 6e,f). IL-10, a cytokine that can function to suppress autoimmune B and T cells, was also elevated in the sera of *lpr/lpr* mice. Introduction of the Fas C194V transgene reduced IL-10 levels similar to those observed in WT mice (Supplementary Fig. 9f). Thus, the resolution of autoimmunity in FasC194V^*lpr/lpr*^ mice is associated with decreased expansion of follicular and extrafollicular helper T cells, but not increased Treg numbers or IL-10 levels.

**Figure 7:**
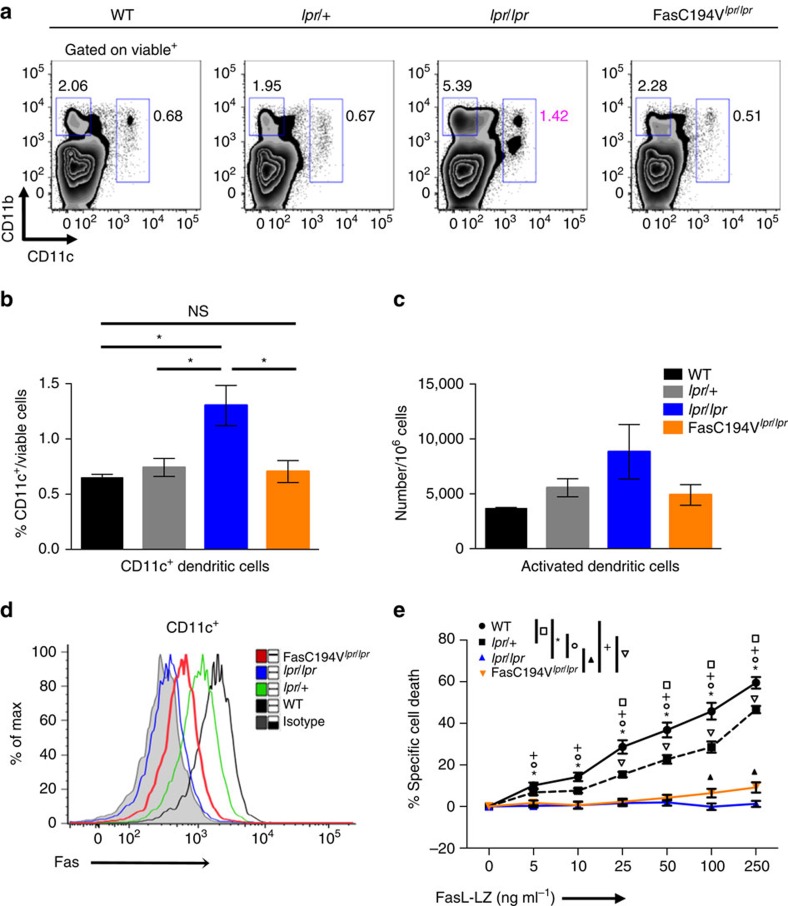
DC apoptosis requires palmitoylation of Fas. (**a**) Spleens from 24-week-old WT, *lpr/*+, *lpr/lpr* and FasC194V^*lpr/lpr*^ mice were isolated and stained for surface markers before analysis by flow cytometry. Data are representative of four independent experiments (*N*=4). Quantitation was performed for CD11c^+^ DCs (**b**) and CD11c^+^CD40^+^CD86^+^MHC-II^+^-activated DCs (**c**) in spleens of the indicated genotypes. Mann–Whitney test was used for statistical analysis. *P≤0.05. NS=Not significant. (**d**) DCs were differentiated from bone marrow of mice (BMDC) of the indicated genotypes for 7 days in RPMI supplemented with GM-CSF and stained for surface Fas before flow cytometric analysis. Cultures were ≥85% CD11c^+^. Data are representative of three independent experiments (*N*=3). (**e**) *In vitro* differentiated BMDCs from WT, *lpr/*+, *lpr/lpr*, or FasC194V^*lpr/lpr*^ mice were cultured in the presence of increasing amounts of FasL-LZ for 8 h before analysis for apoptosis by flow cytometry. Results are compiled from three independent experiments (*N*=3), with compiled data represented as mean ± s.e.m. *P≤0.001, WT versus *lpr/lpr*.^+^*P*≤0.001, WT versus FasC194V^*lpr/lpr*^. Square: *P*≤0.001, WT versus *lpr*/+. Circle: *P*≤0.001 *lpr*/+ versus *lpr/lpr*. Inverted triangle: *P*≤0.001, *lpr/*+ versus FasC194V^*lpr/lpr*^. Filled triangle: *P*≤0.001 *lpr/lpr* versus FasC194V^*lpr/lpr*^. Unpaired *t*-test was used for statistical calculations.

DCs accumulate in *lpr* mice^53^, and DC-specific deletion of the Fas gene or expression of a viral caspase inhibitor permit autoantibody formation and DC expansion^10^,^54^. We therefore investigated the DC compartment in the FasC194V^*lpr/lpr*^ mice at 30 weeks. As previously shown^10^,^53^, we found increased percentages of CD11c^+^ DCs in the spleens of *lpr/lpr* mice compared with WT and *lpr*/+ heterozygous controls. The Fas C194V transgene completely rescued DC accumulation (Fig. 7a,b) and also reduced accumulation of CD86^+^CD40^+^MHC-II^+^ activated DCs (Fig. 7c).

If Fas-dependent DC apoptosis is preserved in FasC194V^*lpr/lpr*^ mice, antigen presentation to T cells and subsequent activation of autoreactive B cells may be restrained, potentially preventing autoantibody formation and the other consequences of Fas deficiency. We investigated whether Fas-induced apoptosis is still functional in FasC194V^*lpr/lpr*^ bone marrow-derived DCs (BMDCs). Similar to the T and B-cell compartments, the Fas C194V transgene was expressed on CD11c^+^ BMDCs (Fig. 7d). However, FasC194V^*lpr/lpr*^ BMDCs were resistant to FasL-induced apoptosis (Fig. 7e). As in other cell types, the Fas C194V transgenic receptor prevented abnormal accumulation of DCs associated with Fas deficiency, despite being defective in inducing cell death.

### ALPS T cells are defective in apoptosis and differentiation

**Figure 8:**
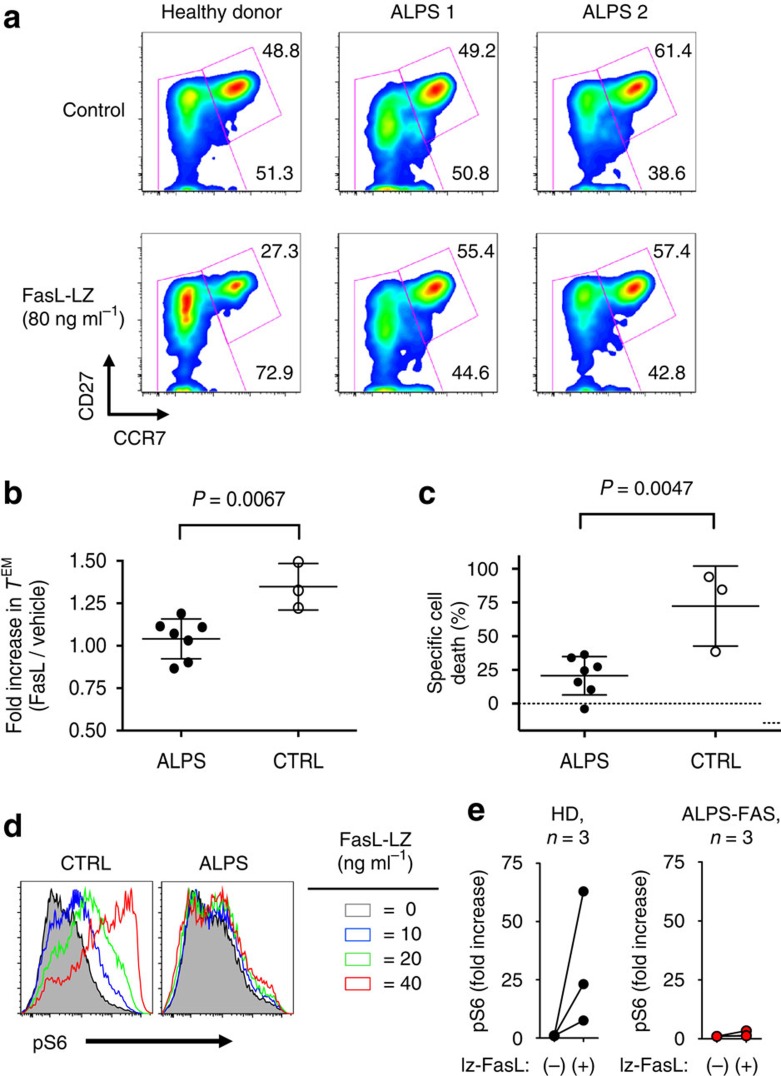
ALPS patient T cells are defective in Fas-induced differentiation and apoptosis. (**a**) Representative data with PBMCs from two unrelated ALPS patients with Fas death domain mutations and a healthy donor control, activated for 7 days with or without FasL-LZ as described in the ‘Methods' section. Differentiation of CD8^+^ T cells was examined by staining for surface CD27 and CCR7. Cells were gated CD3^+^CD4^−^CD8^+^ before analysis of CD27/CCR7 status. (**b**) Summary of fold increase in effector memory T cells by FasL-LZ, in seven ALPS patients (*N*=7) and three normal donor controls (*N*=3) from two independent experiments. Cumulative data are represented as mean±s.e.m. (**c**) Cumulative specific cell death induced by FasL-LZ in cell cultures shown in **b** from two independent experiments. *P*-values are from an unpaired *t*-test, with data represented as mean±s.e.m. (**d**,**e**) Intracellular staining for pS6 was performed on isolated CD8^+^ T cells from three healthy donors (*N*=3) and three ALPS patients (*N*=3), with representative FACS plots (**d**) shown. Summary of data are shown in **e**, with fold increase calculated as pS6 MFI vehicle/pS6 MFI FasL-LZ at 20 ng ml^−1^.

Taken together, the results with FasC194V^*lpr/lpr*^ transgenic mice make the prediction that the lymphadenopathy and accumulation of DN T cells characteristic of the Fas-deficient phenotype should only arise when there are defects in Fas-induced T-cell differentiation, and not just defective apoptosis. To test whether this applies to the human immune system, we investigated the ability of Fas mutants that are defective in cell death to induce non-apoptotic signalling in primary human T cells. In T cells from healthy donors, addition of FasL-LZ to T-cell activation cultures consistently enhanced differentiation of naive T cells into effector memory (CD27^lo^CCR7^lo^) cells, an effect, which was most pronounced in the CD8^+^ T-cell subset (Fig. 8a,b). T cells from ALPS patients harbouring mutations in the Fas death domain are highly resistant to Fas-induced apoptosis due to defective recruitment of FADD and Caspase-8 (refs 4, 11), but whether non-apoptotic functions of Fas are affected in these cells is not known. We obtained T cells from seven unrelated ALPS patients, each with distinct dominant-negative Fas death domain mutations and typical symptoms of ALPS, including resistance of activated T cells to Fas-induced apoptosis (Supplementary Table 1). T cells from these patients were refractory to FasL-induced differentiation (Fig. 8a,b). As expected, ALPS patient T cells were also resistant to induction of apoptosis by Fas Ligand (Fig. 8c). Consistent with our data obtained in T cells derived from *lpr* mice (Fig. 3), ALPS patient cells were unresponsive to FasL-induced phospho-S6 induction, compared with healthy control cells (Fig. 8d,e). These data suggest that in human T cells, a functional death domain is required for both apoptosis and enhanced effector memory T-cell differentiation. Taken together, these data support a model in which Fas-induced T-cell differentiation, not just apoptosis, is necessary to prevent the ALPS phenotype.

## Discussion

The C194V Fas mutant mouse model we describe here confirms the role of palmitoylation in allowing the Fas/CD95 receptor to cluster and efficiently induce apoptosis in primary T, B and DCs. Further, our results define functionally distinct roles for the apoptotic and non-apoptotic functions of Fas in the intact immune system (Supplementary Table 2). Although Fas has long been thought to prevent autoimmunity through inducing apoptosis, our findings with the mutant receptor Fas C194V question this notion. As the Fas C194V receptor is greatly impaired in inducing apoptosis, but can still mediate non-apoptotic signalling, the ablation of the *lpr* phenotype in FasC194V^*lpr/lpr*^ mice suggests that non-apoptotic functions of Fas may play a more important role in prevention of autoimmunity than previously appreciated. We cannot rule out the possibility that the small amount of residual Fas-mediated killing plays a role in preventing the autoimmune phenotype in the FasC194V^*lpr/lpr*^mice. However, several lines of evidence argue against this possibility. T cells from *lpr/+* mice, which express comparable surface levels of Fas as FasC194V^*lpr/lpr*^ T cells retain far higher sensitivity to Fas-induced cell death. The ability of the Fas C194V mutant receptor to mediate accelerated T-cell differentiation shows that receptor levels are high enough to mediate non-apoptotic Fas signalling (Supplementary Table 2). In addition, ALPS patients harbouring dominant-negative Fas mutations in immune cells develop the clinical manifestations of Fas deficiency despite greater residual Fas-induced apoptosis in their T cells^4^. It remains possible that older FasC194V^*lpr/lpr*^ mice than examined in this study may develop autoimmune manifestations of Fas deficiency, but that would still constitute a more than 6 month delay in disease onset, more than would be expected from a receptor with <10% residual apoptotic function. Further studies utilizing Fas-deficient *lpr/lpr* mice on the MRL background will be necessary to determine if the Fas palmitoylation mutant protects from severe autoimmune pathology and nephritis.

If Fas does not protect from autoimmunity through inducing cell death, how does this happen? Our results suggest that non-apoptotic signalling through Fas may promote death of autoreactive T cells indirectly through enhancing differentiation into effector memory cells, a population of lymphocytes known to be more sensitive to many forms of cell death due to both intrinsic and extrinsic cues^25^,^56^. Fas-induced T-cell differentiation may prevent autoantibody formation by promoting B-cell differentiation away from long-lived follicular or extrafollicular helper T cells, which provide help for autoreactive B cells. Indeed, accumulation of T_FH_ and T_EFH_ was reduced in FasC194V^*lpr/lpr*^ mice compared with Fas-deficient *lpr/lpr* mice. Mice engineered to express a ‘soluble only' FasL, which is defective in inducing apoptosis, phenocopy Fas-deficient mice^57^. However, soluble FasL may not be sufficient to drive effector memory T-cell differentiation, as the preparation of FasL that we have used is oligomerized via an isoleucine zipper, and FasL-dependent enhancement of naive T-cell differentiation by memory cells was dependent on cell–cell contact^48^. This suggests that soluble FasL may not be potent enough to enhance T cell differentiation.

Our results highlight the importance of non-apoptotic signalling through Fas in primary immune cells, and prompt renewed examination of the underlying mechanisms. The activation of Akt and subsequent downstream anabolic signalling cascades by FasL co-stimulation in cells expressing the C194V mutant receptor suggest that this pathway is important for non-apoptotic Fas signalling. Whether or not Akt activation is activated directly by Fas or indirectly through other autocrine cytokine loops is an important topic for future investigation. The failure of Fas ligand to enhance differentiation in T cells from ALPS patients harbouring dominant-negative death domain mutations implicates FADD and possibly caspase-8 in non-apoptotic signalling. Of note, T cells from transgenic mice expressing c-FLIP at levels that activate caspase-8 during T-cell activation exhibit increased proliferation, but c-FLIP transgenic mice do not accumulate DN T cells nor develop other features of Fas deficiency^58^.

These results suggest that non-apoptotic signalling through Fas promotes a cell fate (differentiation into effector memory T cells), where cells become sensitive to Fas-induced apoptosis, constituting an elegant feedback system to maintain T-cell homoeostasis during chronic immune stimuli and prevent autoimmunity. Although we found that Fas-induced differentiation into T_EM_ was detrimental to anti-tumour immunity^48^, therapeutic enhancement of T-cell differentiation through non-apoptotic Fas signalling may benefit chronic autoimmune diseases that rely on a self-renewing supply of long-lived central memory T cells. By separating the apoptotic from non-apoptotic functions of Fas, the FasC194V^*lpr/lpr*^ mouse model should aid elucidation of the pathways mediating non-apoptotic signalling, which will inform the design of therapeutic strategies that harness this less-understood function of Fas in the immune system.

## Methods

### Generation of Fas C194V BAC transgenic mice

All animal studies performed were authorized by the NIAMS/NIH Animal Care and Use Committee. Protocol A014-01-01. A Fas C194V BAC transgene was generated by modifying a 210-kb BAC containing the mouse *Tnfrsf6* (Fas) gene (RP23-286N16). Using site-directed mutagenesis, the C194→V mutation (TGC→GTC) was introduced into exon 8, along with silent mutations and deletion of the intron between exons 7 and 8 to direct homologous recombination away from the mutation site and allow for PCR detection of the mutant allele. A construct encoding the altered exons 7–8 was subcloned into pSV1.RecA shuttle vector and transformed into DH10B BAC competent cells expressing the RP23-286N16 BAC. Colony PCR was performed on each clone to assess efficiency of recombination and fully resolved recombinants were picked and plated on LB agar plates containing fusaric acid and chloramphenicol. Clones were screened both by colony PCR and restriction digest to confirm successful recombination according to previously described techniques^59^.

Modified BAC DNA was injected into murine embryos at the blastocyst stage and implanted into female C57BL/6 mice. Pups were screened via Southern Blot using a 500 bp probe generated from purified BAC DNA, digested with the restriction endonuclease AvaII. Seven founders were identified and the two with highest expression of the Fas transgene were crossed with *lpr*/*lpr* mice on the C57BL/6 background. Progeny were assayed for Fas *lpr* and Fas C194V gene expression using primers specific to the *lpr* mutation^60^ or primers targeting exonic regions flanking the C194V mutation and putative deleted intron between exon 7 and 8 (Forward: aaatggcgggaatgagaggacag. Reverse: tgtgcagctcagtagagagcggg).

For Fas *lpr* copy number determination, primers specific to the Etn translocation were used (Forward: acagcatagattccatttgctgct. Reverse: caaattttattgttgcgacacca^60^). For copy number determination of Fas C194V, specific primers to the manipulated C194V sequence were designed so that amplification could only be obtained if the mutation was present (Forward: gtgtgaacatggaacccttgag. Reverse: ctTctCttccagACTttCctCttG. Capital letters are introduced silent mutations). GAPDH was run as the reference gene (Forward: catgttccagtatgactccactc. Reverse: ggcctcaccccatttgatgt.), with known heterozygote and WT (no transgene) DNA as control. The ratio of the BAC transgene relative to GAPDH reference was calculated as previously described^61^.

### Fas-ligand LZ preparation

FasL-LZ was generated through overexpression of the extracellular domain of FasL, fused to a FLAG tag and an isoleucine zipper motif for receptor self-oligomerization, in HEK293T cells. Transfected cell supernatants were collected and protein was purified over magnetic beads conjugated to an antibody to the FLAG tag (anti-FLAG M2 beads, Sigma), with quantitation performed by ELISA (R&D Systems).

### Flow cytometry

For flow cytometric analysis of *ex* vivo or *in* vitro cells from mouse and human samples, cells were washed once in ice-cold Hanks' buffered saline solution (HBSS). A total of 5 × 10^6^ cells were stained in HBSS at a concentration of 20 × 10^6^ cells per ml for 30 min at room temperature in the presence of fixable viability dye and appropriate antibody cocktails, and subsequently washed once in cold HBSS supplemented with 0.5% BSA before analysis on an SORP LSRII Fortessa or a SORP FACS Canto flow cytometer (BD Biosciences). Gating for cell populations of interest were performed as shown in Supplementary Fig. 10. Antibodies were used at the indicated dilutions from the following sources: mouse anti-TCRβ (H57-597; 1:200 dilution), CD4 (GK1.5; 1:400), CD8 (53-6.7; 1:200), CD11b (M1/70; 1:300), CD11c (N418; 1:200), CD19 (eBio1D3; 1:500), CD21/35 (eBio4E3; 1:200), CD23 (B3B4; 1:400), CD40 (1C10; 1:200), CD44 (IM7; 1:200), CD45 (30-F11; 1:1,000), CD62L (MEL-14; 1:800), CD86 (GL1; 1:600), FoxP3 (FJK-16 s; 1:200), GL-7 (GL-7; 1:200), GR-1 (RB6-8C5; 1:500), IgD (11-26; 1:200), IgM (II-41; 1:200), MHC-II I-A^b^ (AF6-120.1; 1:500), PD-1 (J43; 1:200), CXCR4 (2B11; 1:100), CXCR5 (SPRCL5; 1:100), TCR Vβ8 (KJ16; 1:500), human anti-CD4 (OKT4; 1:200), CD8 (HIT8a; 1:200) and CD27 (LG.7F9; 1:100) were all from eBioscience. TCR Vβ6 (RR4-7; 1:500 dilution), PSGL-1 (2PH1; 1:200), B220 (RA3-6B2; 1:1,000) and Fas (DX-2; 1:100) were obtained from BD Pharmingen. Anti-human CCR7 (150503; 1:25 dilution) was from R&D Systems. Annexin V (1:120), DAPI (1:1,000) and Live/Dead Blue Fixable Viability Dye (1:500) were from Molecular Probes/Invitrogen. For intracellular staining, cells were fixed in 3.7% paraformaldehyde for 15 min at 37 °C, washed twice and permeabilized in 90% ice-cold methanol for 20 min, −20 °C. Cells were washed twice with FACS buffer and stained for 1 h at room temperature with anti-pS6 S235/236 FITC (clone D57.2.2E; 1:100 dilution; Cell Signaling Technologies).

For human pS6 and surface staining, cells were harvested and washed with ice-cold PBS, stained with fixable FITC Live/Dead (1:500; Invitrogen) at 4 °C in PBS, washed with 4 °C FACS Buffer (PBS with 2.5% BSA and 0.02% Sodium Azide), then stained with desired surface antibody-fluorochrome conjugates in FACS Buffer at 4 °C. Cells were then washed with 4 °C FACS Buffer incubated in pre-warmed 37 °C 1 × Lyse/Fix Buffer (BD Biosciences) for 10 min, washed with room temperature FACS Buffer and pellets were resuspended in pre-chilled Perm Buffer III (BD Biosciences). Cells were then incubated at −80 °C for at least 2 h, resuspended in room temperature FACS Buffer and incubated with anti-pS6 (1:100 dilution) for 1-hour in FACS Buffer, washed and analysed via Flow Cytometry. All flow cytometric data were acquired using a SORP LSRII Fortessa flow cytometer (BD Biosciences).

### Fluorescent resonance energy transfer

Full-length human or Fas lacking the death domain (Fas ΔDD) were cloned into pEYFP-N1 (Clontech), with the C199V palmitoylation mutation introduced via site-directed mutagenesis. Human FADD death domain (FADD DD) was cloned into pECFP-N1 (Clontech). Each construct was transfected into HEK293T cells using Fugene 6 HD (Promega). Cells were analysed 48 h post-transfection by flow cytometric detection of FRET^62^,^63^ using a SORP LSRII Fortessa flow cytometer (Becton Dickinson) with 447/488nm dual laser excitation. FRET data are shown for cells gated to comparable expression of the CFP and YFP fusion proteins.

### Cell isolation and death assays

Murine CD4^+^ T cells were isolated from 6 to 12 week-old WT (C57BL/6), *lpr/+* heterozygous, *lpr/lpr* homozygous or FasC194V^*lpr/lpr*^ mice via magnetic separation (EasySep Mouse CD4 Isolation Kit, StemCell Technologies). Cells were cultured on plate-bound anti-CD3/CD28 (5 μg ml^−1^) for 72 h, 37 °C and subsequently cultured in RPMI media supplemented with recombinant IL-2 (100 U ml^−1^) for 5 days before cell assays.

To assess Fas-induced cell death, freshly isolated *ex vivo* or *in vitro-*activated cells were incubated with increasing levels of FasL-LZ for 6–8 h, 37 °C and analysed using a multiparameter flow cytometry assay as previously described^25^,^64^ using Annexin V and Live/Dead Viability Dye as readout for cell death. Briefly, cells were plated at a density of 2 × 10^6^ cells per ml in complete RPMI media supplemented with 10% FBS and 100 U ml^−1^ recombinant IL-2 (NIH Biorespository, Frederick, MD, USA). Cells were cultured with increasing amounts of FasL-LZ for 6–8 h at 37 °C and stained in a multiparameter format with the following antibodies: mouse anti-TCRβ (H57-597), CD4 (GK1.5), CD8 (53-6.7), CD44 (IM7), CD62L (MEL-14) (all from eBioscience); mouse anti-B220 (RA3-6B2, BD Biosciences) using the concentrations described above. Cells were gated on TCRβ^+^CD4^+^CD44^+^ for *in vitro*-activated cells and TCRβ^+^B220^−^CD4^+^CD44^hi^CD62L^lo^ for fresh *ex vivo* cells. Specific cell death was calculated using the formula ((1−% live-treated cells)/(% live-untreated cells)), wherein Annexin^−^ cells are considered live cells. For assaying restimulation-induced cell death (RICD), activated and cultured cells were incubated on increasing concentrations of plate-bound anti-CD3 (clone 145-2C11; BioXCell) for 8 h, 37 °C and stained with Annexin/DAPI or Annexin/Live/Dead in a multiparameter format. Cells were gated CD4^+^CD44^+^ for analysis.

B cells were isolated from spleens of 6–12 week-old WT, *lpr/+*, *lpr/lpr* or FasC194V^*lpr/lpr*^ mice via magnetic separation (EasySep Mouse B Cell Isolation Kit, StemCell Technologies) and cultured 72 h in RPMI supplemented with LPS (LPS from *E.coli* O111:B4, Sigma) before treatment with increasing amounts of FasL-LZ and assessed for cell death as described above.

For BMDCs, bone marrow was isolated from femurs of the above genotypes and differentiated in RPMI supplemented with murine GM-CSF (10 ng ml^−1^; Peprotech) for 7 days. Non-adherent cells were isolated and stained for CD11c to assess purity, with cultures achieving ≥85% CD11c^+^. 7 day cultures were stimulated with increasing amounts of FasL-LZ and assessed for cell death as described above.

### Induction of T-cell deletion by SEB

For induction of selective deletion of Vβ8^+^CD4^+^ T cells, 8–10-week-old C57BL/6 (WT), *lpr/lpr* or FasC194V^*lpr/lpr*^ mice were injected intraperitoneally with PBS or 20 μg of purified Staphylococcal Enterotoxin B (SEB; Toxin Technology) serially on day 0, 1, 2 and 3. Spleens were harvested on day 10 and stained for T-cell and Vβ TCR markers (Vβ6 and Vβ8) and analysed by flow cytometry.

### Autoantibody quantitation

Antibody titres to nuclear proteins (anti-nuclear antibodies, ANA) or double-stranded DNA (dsDNA) were quantified by ELISA (Alpha Diagnostics). Data were shown as an autoantibody index, determined by calculating the optical density (OD)+2 s.ds of the control samples (positive index) and dividing each sample net OD by the positive index. Values >1 were considered positive.

### Fas lipid raft localization and palmitoylation

HEK293T cells were transfected with constructs expressing human Fas or palmitoylation-deficient C199V Fas, fused to EYFP on the C terminus. 24 h post-transfection, cells were pulsed with azide-linked palmitic acid (palmitic acid-azide, 100 mM; Invitrogen) for 24 h at 37 °C. Cells were lysed in lysis buffer (30 mM Tris–HCl, pH 8, 150 mM NaCl, 10% v/w glycerol) containing 1% Triton X-100 and debris were cleared via centrifugation. Lysate protein content was quantified using Bicinchoninic Acid (Micro BCA Kit, Pierce). Overall, 1 mg total protein was isolated and biotin labelling of palimitic acid was performed using the Click-iT Protein Reaction Kit and amide-linked biotin (Invitrogen). To reduce background levels of amide-linked biotin, protein was precipitated using chloroform/methanol extraction and resolubilized in 50 mM Tris–HCl, pH 8 containing 1% SDS. Concentrated protein was then diluted 1:2 with lysis buffer and immunoprecipitated for 16 h at 4 °C with streptavidin covalently coupled to agarose beads (Pierce Biotechnology). Beads were washed and eluted using protein loading buffer containing 100 μM dithioltheritol (Bio-Rad). Immunoprecipitated proteins were run on 4–12% Bis–Tris acrylamide gels in MOPS running buffer (Invitrogen), transferred to PVDF and immunoblotted for Fas (anti-Fas C-20, Santa Cruz Biotechnology) or YFP (anti-GFP, Roche).

For lipid raft fractionation, Optiprep density ultracentrifugation was performed as previously described^40^ on HEK293T cells transfected as above. Cells were also transfected with a construct expressing MyrPalm fused to CFP to identify lipid raft microdomains (gift from Roger Tsien^65^).

All original, uncropped Western blots are shown in Supplementary Fig. 11, with red boxed regions used in the final figures.

### FasL-induced T-cell differentiation assays

Naive CD4^+^ T cells (CD62^hi^CD44^lo^) were isolated from lymph nodes and spleens of 6–11-week-old WT, *lpr/lpr*, or FasC194V^*lpr/lpr*^ mice via magnetic separation (EasySep Naive CD4 Isolation Kit, StemCell Technologies). Purified naive cells were stimulated with 2 μg ml^−1^ plate-bound anti-CD3 and 1 μg ml^−1^ soluble anti-CD28 antibodies (BD Biosciences) supplemented with 100 U ml^−1^ recombinant IL-2, in the absence or presence of increasing amounts of FasL-LZ for 48 h. Activated cells were then removed from anti-CD3/28 stimulation, transferred to culture media containing IL-2 in the presence or absence of FasL-LZ and cultured for 72 h. Cells were stained for activation markers (CD62L, CD44) as well as cell death (Annexin V), and flow cytometry was performed to determine effector cell phenotype (a measure of precocious differentiation) as well as measure apoptosis.

Frozen PBMCs from seven unique ALPS patients and two healthy control relatives were thawed and rested in RPMI supplemented with IL-2 for 16 h. Total T cells were isolated by magnetic separation (EasySep Human T Cell Enrichment Kit, StemCell Technologies). T cells were activated for 48 h on anti-CD3/CD28-coated plates (1 μg ml^−1^) supplemented with 100 U ml^−1^ IL-2 in the presence or absence of 80 ng ml^−1^ FasL-LZ. Activated T cells were expanded with IL-2 for an additional 5 days in the presence or absence of FasL-LZ. Cells were stained for surface markers CCR7 and CD27, as well as Annexin V and Live/Dead Blue Viability Dye before assessment via flow cytometry (gated CD3^+^CD4^−^CD8^+^). All samples were obtained with prior written consent and approved by the NIAID IRB, NIH under protocol 93-I-0063, NCT00001350.

### PALM imaging

mEos2 in pRSETa vector^66^ was a kind gift from Loren Looger. mEos2-N1 was generated by amplifying mEos2 with a 5′ primer encoding an Age1 site and a 3′ primer encoding a BsrG1 site and inserting the digested PCR product into an Age1/BsrG1 digested pEGFP-N1 (Clontech Laboratories) vector. To generate Fas-mEos2 fusion constructs, WT Fas was cloned into pECFP-N1 (Clontech) modified to contain the TNFR2 signal sequence and HA tag upstream of the multiple cloning site. The C199V mutation was introduced via site-directed mutagenesis. CFP was removed and mEos2 was introduced to the construct by restriction digest (AgeI/BsrGI).

PALM experiments were carried out by transiently expressing mEos2-lableled human WT, palmitoylation-deficient C199V Fas or WT Fas lacking the death domain (Fas ΔDD) in COS-7 cells. COS-7 cells were maintained in Phenol Red-free Dulbecco's Modified Eagle Medium (Cellgro) supplemented with 10% fetal bovine serum, 1 mM sodium pyruvate, 100 U ml^−1^ penicillin, 100 U ml^−1^ streptomycin and 2 mM glutamine (Invitrogen). For PALM experiments, cells were plated in fibronectin (Sigma) coated pre-cleaned coverslips^67^. Approximately 24 h after plating, cells were transfected with appropriate plasmid constructs using Fugene 6 (Promega). Cells were imaged at 37 °C in pH-independent DMEM (Invitrogen) supplemented with 2% fetal bovine serum 24 h post-transfection.

PALM imaging of live cells was performed on a Zeiss Elyra PS.1 PALM microscope (Carl Zeiss, Thornwood, NY) with a 100 × 1.46 NA objective. Images of live cells were collected within an 81.9 × 81.9 μm^2^ area with an exposure of 50 ms. mEos2 was either activated and excited simultaneously with a single 543 nm laser, or was activated with a combination of 405/543 nm laser and excited with 543 nm laser. The laser intensities were chosen to maintain a sparse population of activated molecules in each image frame to allow precise localization. Single molecule peaks were identified and localized using Zeiss Zen software (Carl Zeiss, Thornwood, NY, USA). Each composite PALM image comprised of single molecule localizations from 3,000 to 5,000 successive image frames. Only single molecules localized with a precision <35 nm were included in the composite images. Each localized molecule was rendered as a Gaussian of s.d. equal to its localization precision and with amplitude normalized to 1 when integrated over the entire xy space. The final PALM composite image comprised of the sum of all the rendered molecules. Images are rendered with pseudocolor coding of probability density of fluorescent molecules, with brightness proportional to the probability of finding a molecule at a given location. The image rendering was performed using custom software written in IDL.

### Cytokine and immunoglobulin measurement and analysis

Serum from WT, *lpr/lpr*, or FasC194V^*lpr/lpr*^ mice at least 24 weeks of age was analysed for protein levels of the cytokines IL-6, IL-10, TNF, IL-17A, IL-17F and IL-21 via Luminex bead-based protein detection assay (Bio-Rad) according to the manufacturer's protocol. Protein concentrations were detected and calculated on a Luminex MagPix and Bio-Plex Manager 6.0 (Bio-Rad) and shown as absolute values of pg ml^−1^. Serum immunoglobulin levels were detected via Milliplex Mouse Immunoglobulin Isotyping Panel according to the manufacturer's instructions, and detected as above.

### Mouse tissue histology

Twenty-eight-week-old mice were anesthetized by i.p. administration of ketamine/xylazine mixture (1 ml ketamine (100 mg ml^−1^), 0.5 ml xylazine (20 mg ml^−1^) and 8.5 ml PBS). Mice were bled by cardiac puncture, and subsequently perfused to remove blood by inserting a butterfly needle into the left ventricle and passing 15 ml PBS through the vasculature. Tissues were either fixed in formalin or embedded in OCT and snap-frozen in an isopentane bath cooled with dry ice. For frozen tissues, 7 μm sections were cut using a cryostat (Leica CM1850) cooled to −21 °C and mounted on SuperFrost plus slides (Fisher). All subsequent steps were carried out at room temperature. Sections were dried overnight and fixed in acetone for 10 min. For immunohistochemistry, sections were blocked with 2.5% normal goat serum (Vector Laboratories) in PBS for 30 min before staining with an F4/80 antibody (Cl:A3-1) or CD3 antibody (KT3) in PBS with 0.05% Tween 20 (Sigma) for 2 h. Slides were incubated with Peroxidase Suppressor (Thermo Fisher) for 15 min. Sections were incubated with ImmPress anti-rat Ig peroxidase (Vector Laboratories) for 30 min and developed using DAB peroxidase substrate kit (Vector Laboratories) for 2–5 min. Tissue was counterstained with hematoxylin (Vector Laboratories) for 3–5 min, washed with 2% glacial acetic acid and blued with 0.45% NaOH in 70% ethanol for 30 s. For immunofluorescence, sections were blocked in 10% normal serum of the secondary antibody host in PBS for 30 min before staining with a FITC-conjugated C3 (RmC11H9) or IgG (Poly4503) antibody for 45 min. All slides were mounted using VectaMount AQ (Vector Laboratories). All images were acquired with a BZ-9000 microscope and analysed with the BZ-II Analyzer software package (Keyence). Immunofluorescence was quantified using ImageJ software. Immunohistochemical image backgrounds were adjusted to white using Adobe Photoshop.

### Measurement of albumin/creatinine ratio

Urine from age-matched WT, *lpr/lpr*, or FasC194V^*lpr/lpr*^ mice were measured for albumin and creatinine by enzyme immunoassay (Alpha Diagnostics and R&D Systems, respectively).

### Data availability

The data that support the findings of this study are available from the corresponding author upon request.

## Additional information

**How to cite this article:** Cruz, A. C *et al*. Fas/CD95 prevents autoimmunity independently of lipid raft localization and efficient apoptosis induction. *Nat. Commun.* **7,** 13895 doi: 10.1038/ncomms13895 (2016).

**Publisher's note:** Springer Nature remains neutral with regard to jurisdictional claims in published maps and institutional affiliations.

## Supplementary Material

Supplementary Information Supplementary Figures and Supplementary Tables
