# Supplementary material for: Fas/CD95 prevents autoimmunity independently of lipid raft localization and efficient apoptosis induction
Source: Nat Commun. 2016 Dec 23;7:13895. doi: 10.1038/ncomms13895 (PMC5196435; doi:10.1038/ncomms13895)
Supplement: Supplementary Information — Supplementary Figures and Supplementary Tables [file ncomms13895-s1.pdf]

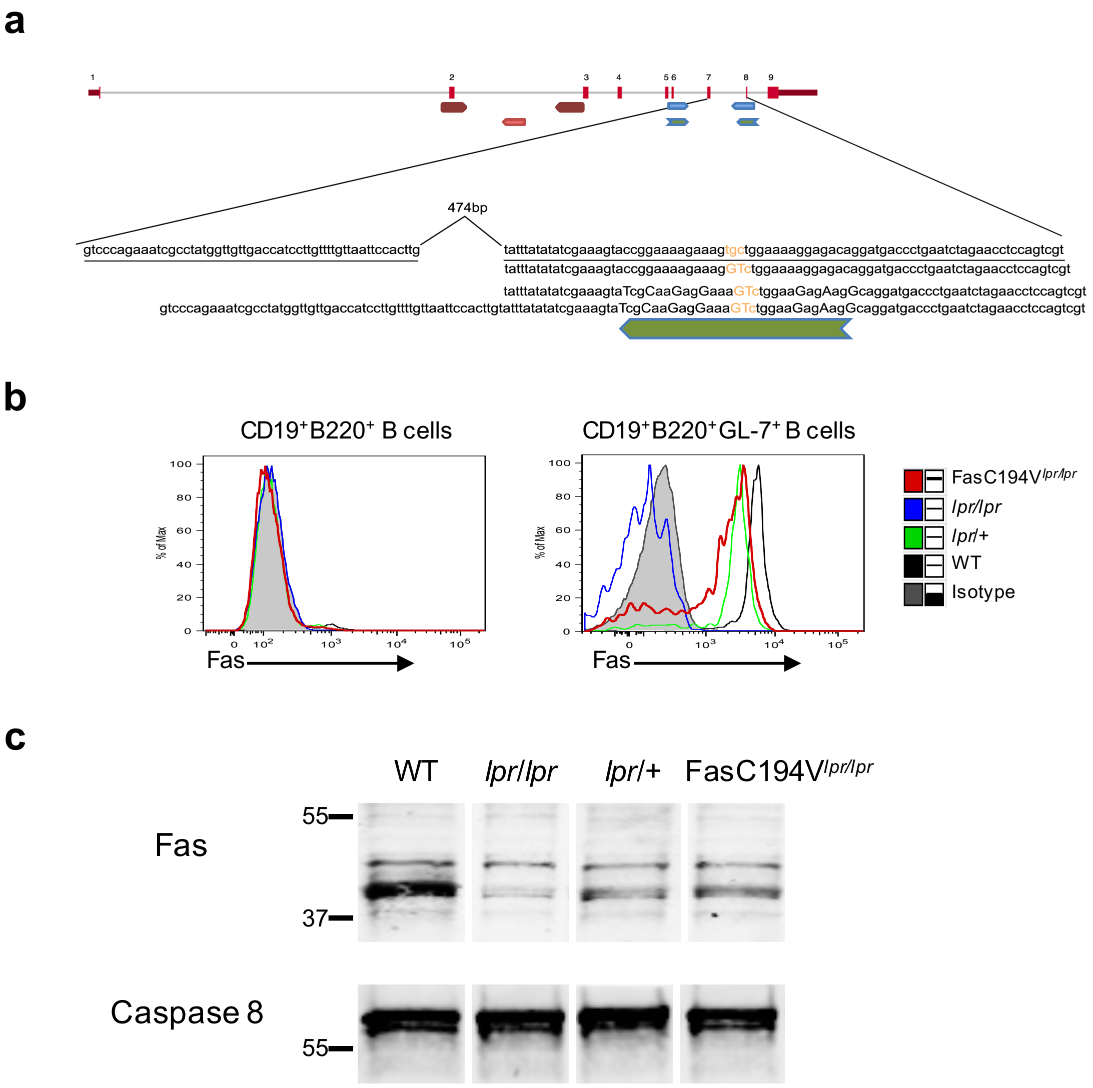

Supplementary Figure 1: **Generation of Fas C194V mutant BAC transgenic mice.** **(a)** Exons 6-8 of genomic murine Fas were isolated via PCR from a Bacterial Artificial Chromosome (BAC) containing the full Fas locus. Site-directed mutagenesis to insert the C194→V mutation was performed and the flanking intron was removed. Recombineering was performed to introduce the modified exons 6-8 into the BAC. **(b)** Flow cytometry was performed on *ex vivo* resting total ($CD19^+B220^+$) and germinal center-type ($CD19^+B220^+GL\text{-}7^+$) B cells of 10 week-old wild-type (WT), *lpr*/+, *lpr/lpr* or $FasC194V^{lpr/lpr}$ mice. Grey shaded: isotype control; Black: WT; Green: *lpr*/+; Blue: *lpr/lpr*; Red Bold: $FasC194V^{lpr/lpr}$. **(c)** Immunoblot analysis of Fas levels on activated T cells. Lysates were immunoblotted for murine Fas or Caspase-8 (loading control). Data are representative of two independent experiments (N=2).

a

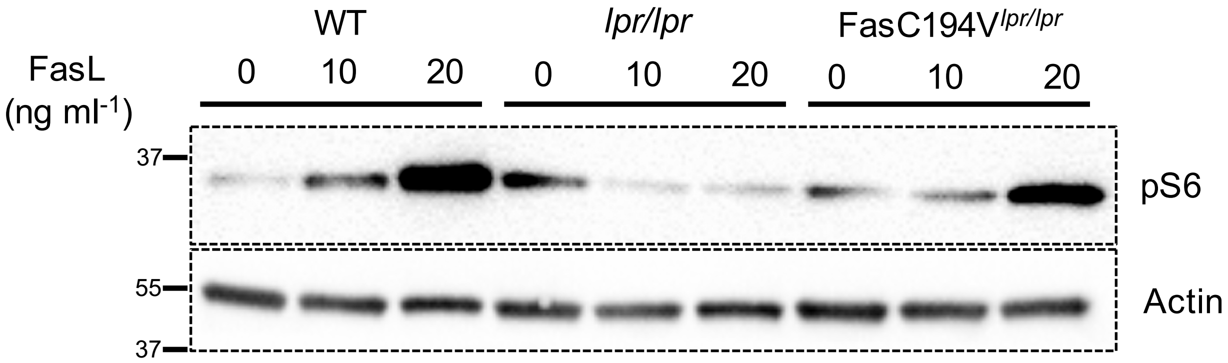

b

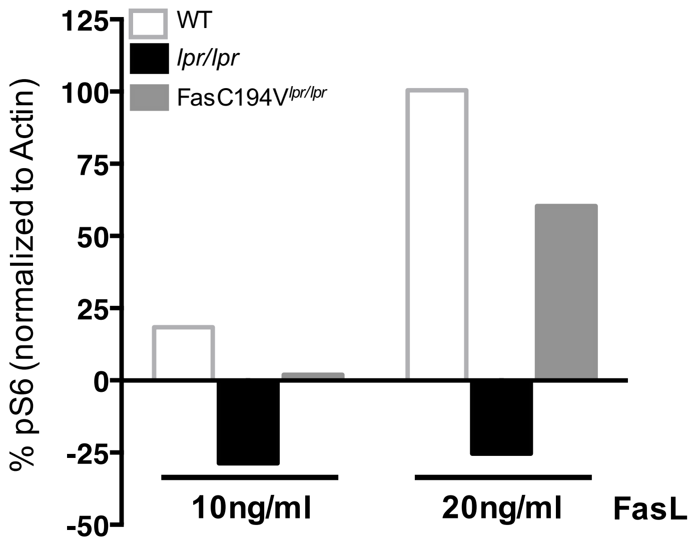

Supplementary Figure 2: **Sustained phospho-S6 activation mediated by the Fas C194V receptor (a)** Immunoblot analysis for pS6 and actin loading control was performed on day 6 precocious differentiated CD4$^+$ T cells that were treated or untreated with FasL at the indicated doses. Densitometry of pS6 levels normalized to actin is indicated below **(b).** The results are representative of two independent experiments (N=2).

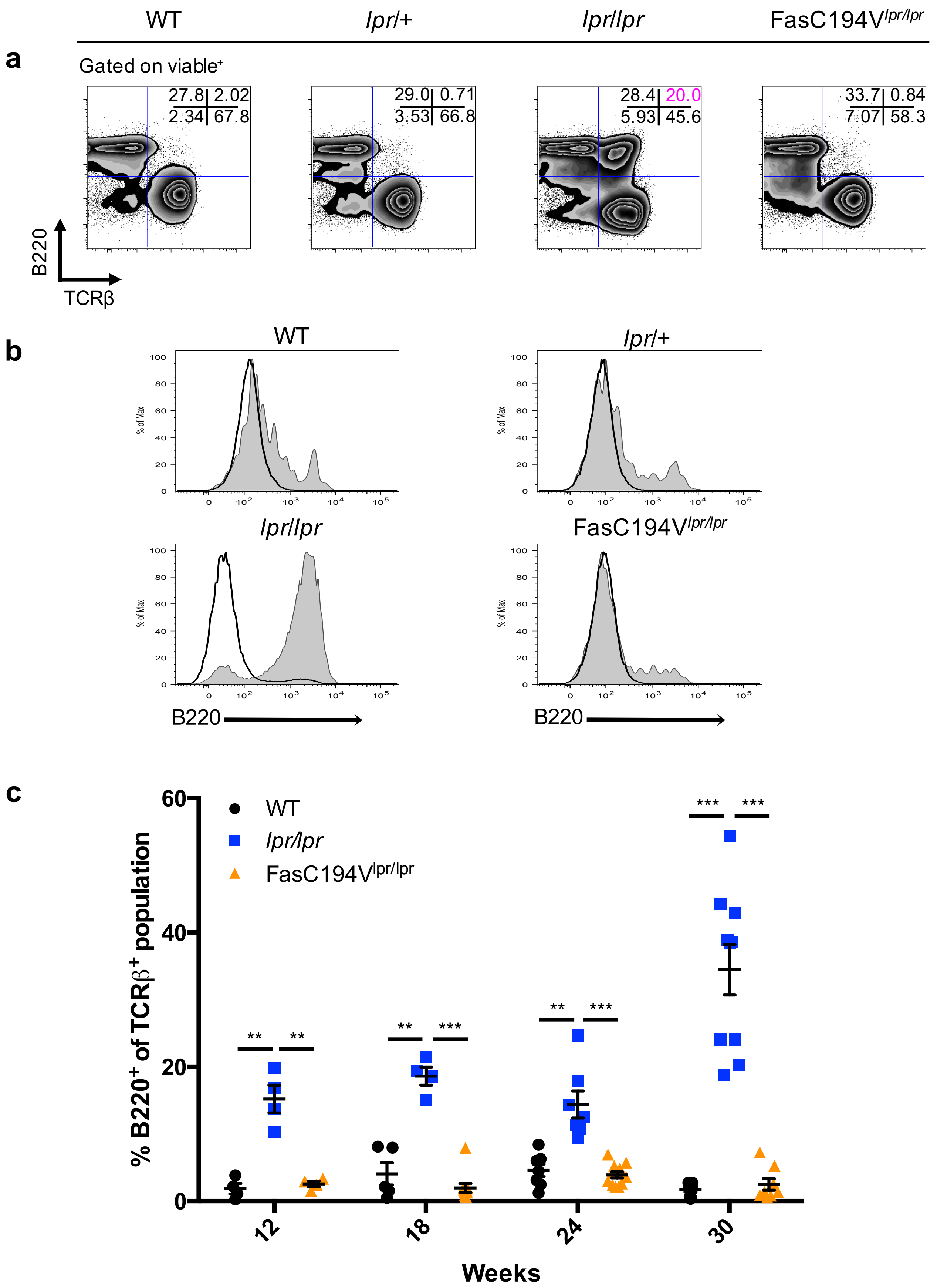
WT
lpr/+
lpr/lpr
FasC194V lpr/lpr
a
Gated on viable+
27.8 2.02 2.34 67.8
29.0 0.71 3.53 66.8
28.4 20.0 5.93 45.6
33.7 0.84 7.07 58.3
B220
TCRβ
b
WT
lpr/+
lpr/lpr
FasC194V lpr/lpr
% of Max
B220
c
WT
lpr/lpr
FasC194V lpr/lpr
% B220+ of TCRβ+ population
Weeks
12
18
24
30

Supplementary Figure 3: TCRβ$^+$B220$^+$ DN T cells do not accumulate in FasC194V$^{lpr/lpr}$ mice. **(a)** Spleens of age-matched WT, *lpr*/+, *lpr/lpr*, or FasC194V$^{lpr/lpr}$ mice were isolated, stained for the indicated markers and analyzed via flow cytometry. Cells were gated on the viable population and representative of at least 3 independent experiments. Mice were approximately 30 weeks of age. **(b)** Histograms of B220 expression on TCRβ$^+$CD4$^-$CD8$^-$ DN T cells (grey shaded) or TCRβ$^+$ cells positive for CD4 or CD8 (black line) from the spleens of the indicated genotypes. Cells were gated on the viable lymphocyte population. **(c)** Analysis of percentage of B220$^+$ population within TCRβ$^+$ cells. Spleens from WT, *lpr/lpr*, or FasC194V$^{lpr/lpr}$ mice of the indicated ages were stained and analyzed as above. % B220$^+$ were determined within the viable cell population gated for TCRβ$^+$, with each data point representing a single mouse. Data are compiled from at least 2 independent experiments of each age (n ≥ 5 for each genotype). Mann-Whitney test was used for statistical analysis. ** $p \leq 0.01$; *** $p \leq 0.001$.

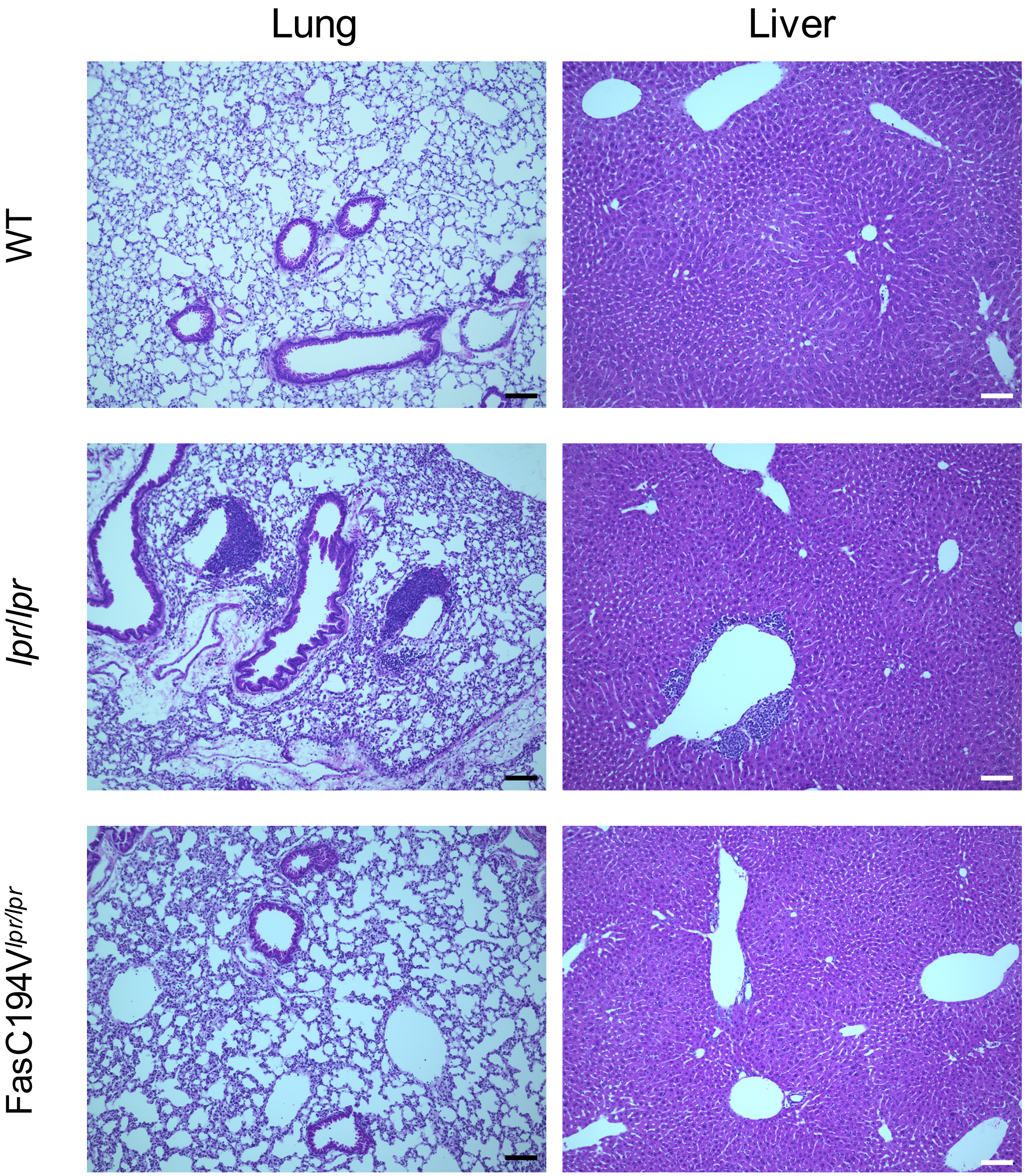

Supplementary Figure 4: Fas C194V reduces lymphoid infiltrates in the lung and liver. H&E sections from lung **(a)** and liver **(b)** of age-matched WT, *lpr/lpr*, or FasC194V$^{lpr/lpr}$ mice. Images are representative of minimum 4 mice per genotype. Mice were 28 weeks of age at time of harvest. Scale bar: 100µm.

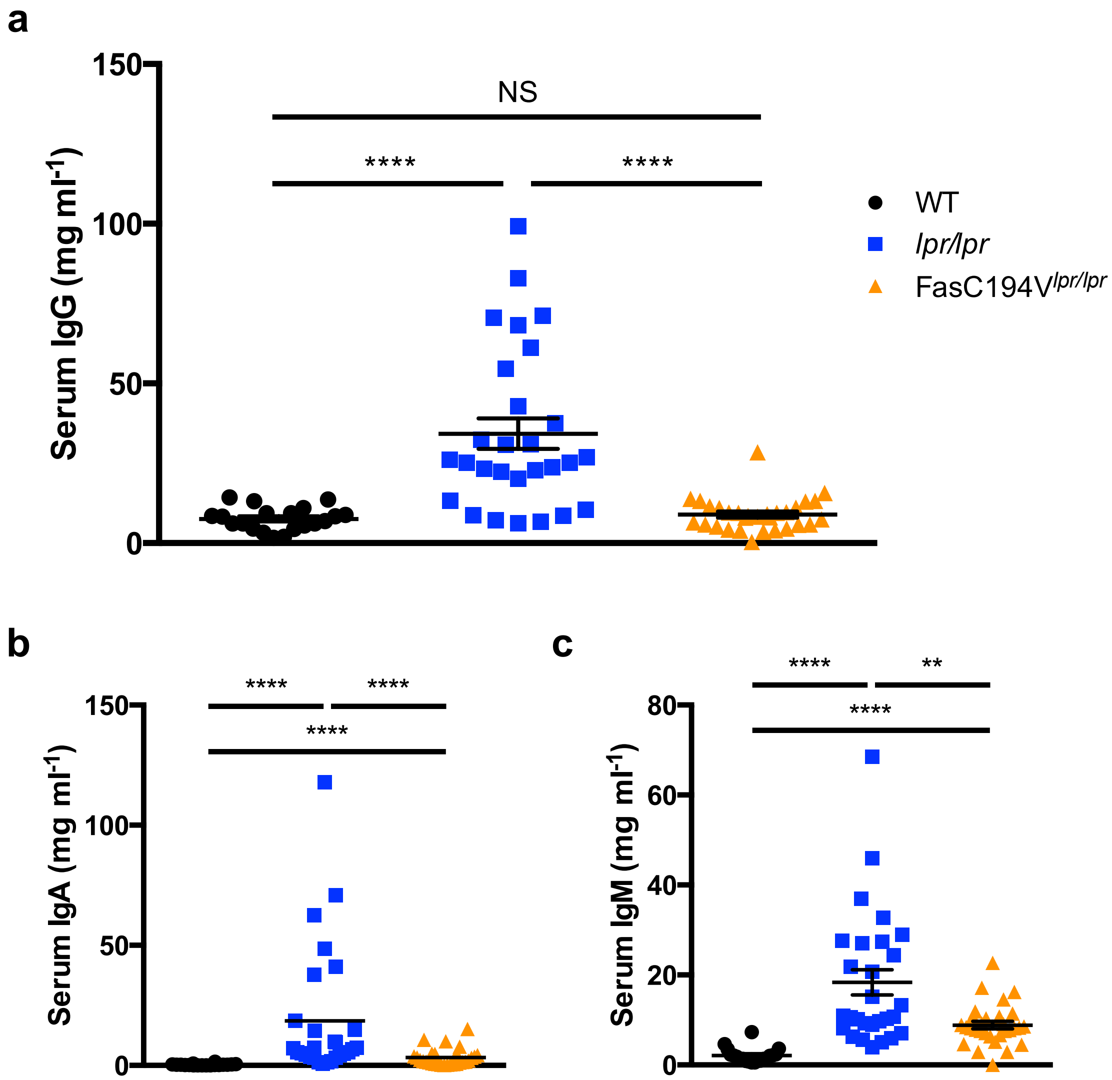

Supplementary Figure 5: Hypergammaglobulinemia is diminished in FasC194V*lpr/lpr* mice. Serum was collected from mice of the indicated genotypes and analyzed for immunoglobulin subtypes via bead-based protein detection assay. Total serum IgG **(a)**, IgA **(b)**, and IgM **(c)** were measured in a minimum of 20 mice per genotype ($n \geq 20$). Mice were at least 28 weeks of age at the time of serum collection. Mann-Whitney was used for statistical analyses. ** $p \leq 0.01$; **** $p \leq 0.0001$; NS = Not significant.

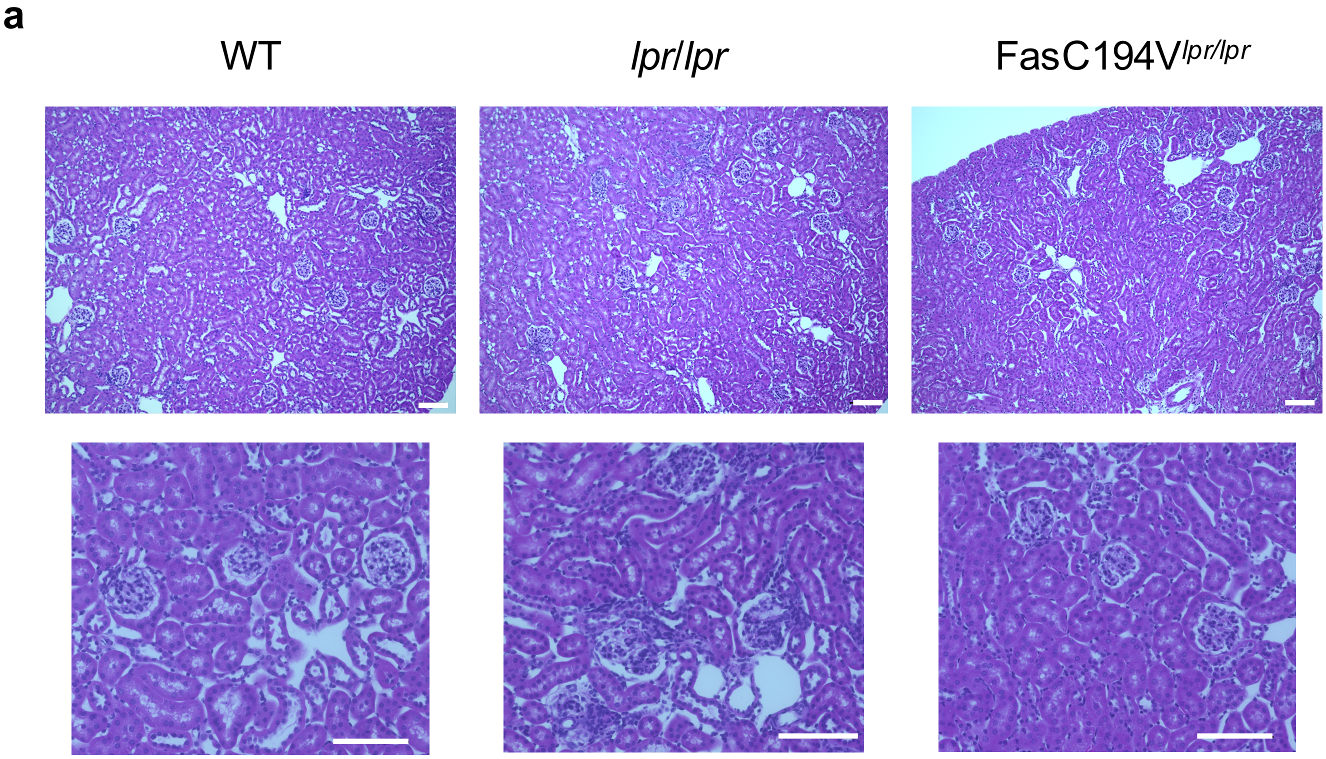
a
WT
lpr/lpr
FasC194V lpr/lpr

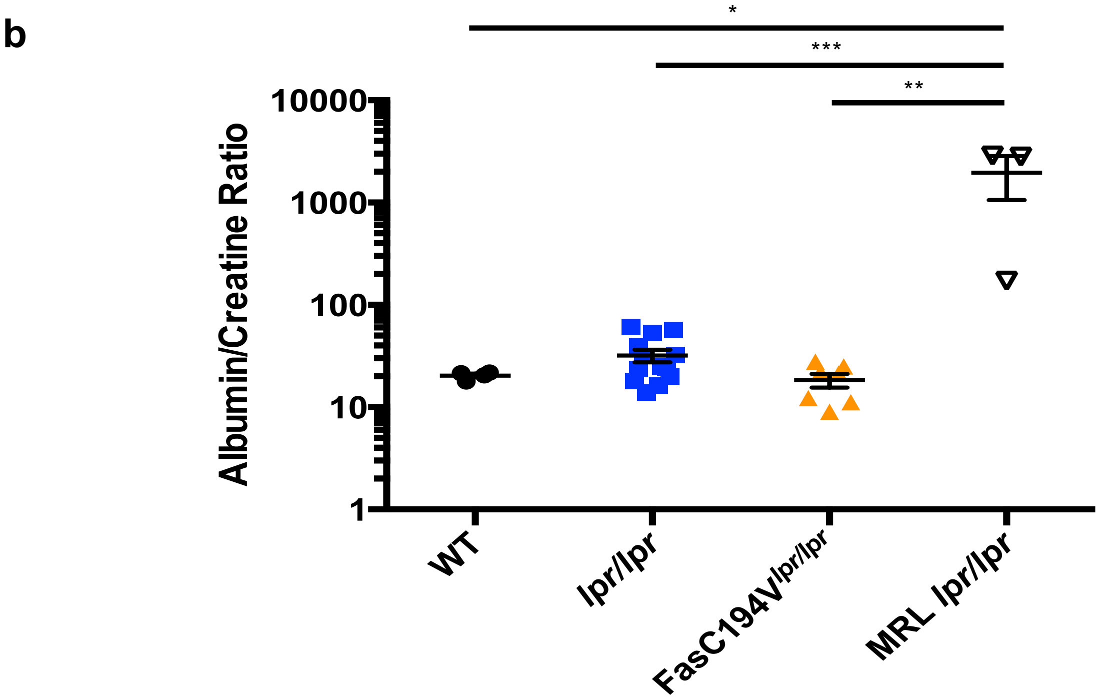
b
*
***
**
Albumin/Creatine Ratio
10000
1000
100
10
1
WT
lpr/lpr
FasC194V lpr/lpr
MRL lpr/lpr

Supplementary Figure 6: Minimal kidney pathology and proteinurea in C57BL/6 *lpr/lpr* mice. **(a)** H&E-stained sections of kidney harvested from 8 month-old age-matched WT, *lpr/lpr*, or FasC194V$^{lpr/lpr}$ mice. Images are representative from an individual mouse (n ≥ 4 for each genotype). Higher magnification images to show glomeruli from indicated mouse genotypes are shown in lower panels. **(b)** Urine was harvested from age-matched 8 month-old mice of the indicated genotypes and analyzed for both albumin and creatinine levels. Data is shown as the ratio of albumin to creatinine within an individual sample, with each data point representing a single mouse. Data are cumulative of 2 independent experiments (n ≥ 5 for each genotype). Ratio of urine albumin to creatinine for 16 week-old *lpr/lpr* mice on the MRL background is shown as a positive control. Unpaired t-test was used for statistical analyses. * $p \leq 0.05$; ** $p \leq 0.01$; *** $p \leq 0.001$ versus positive control. Scale bar: 100μm.

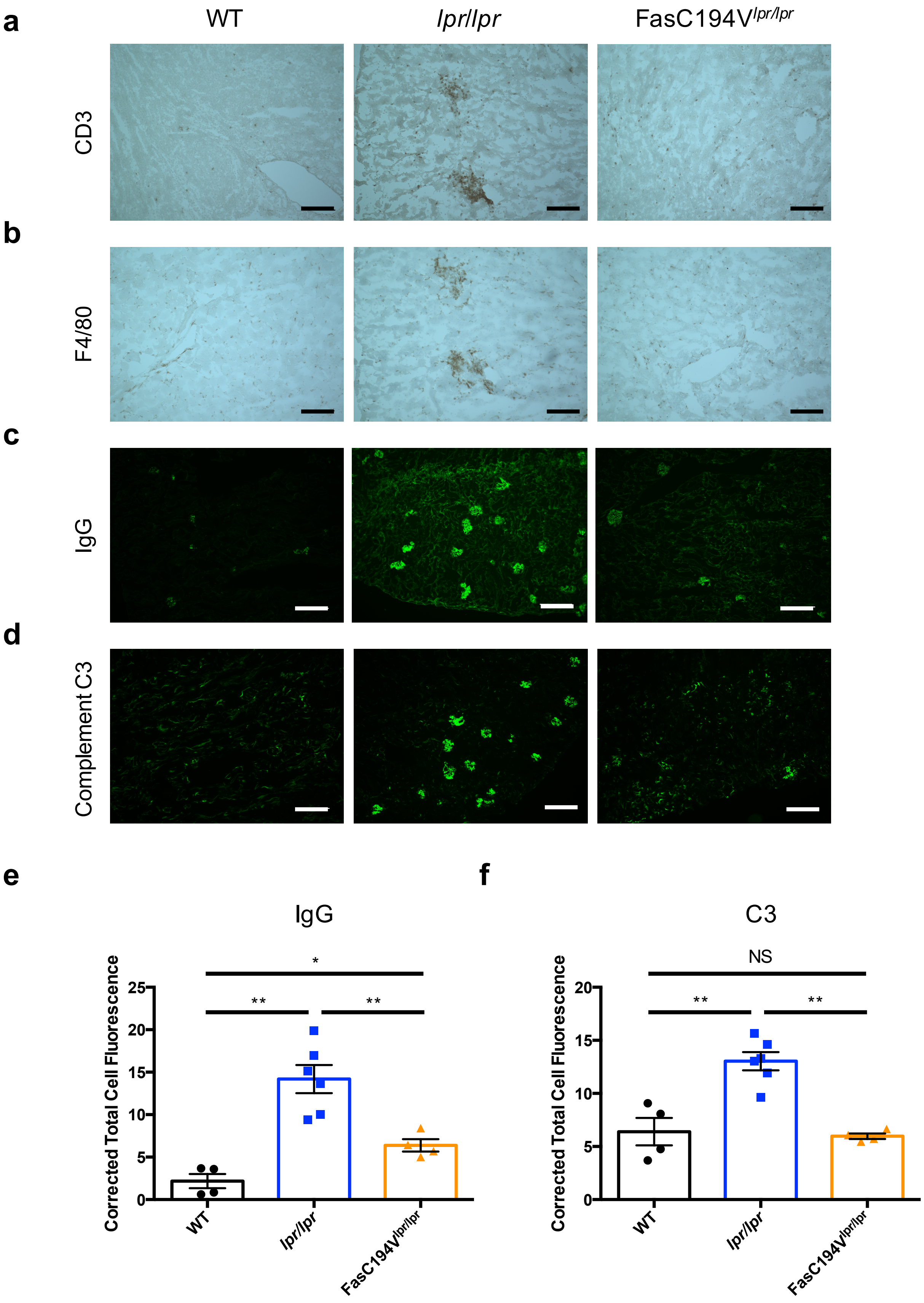
a
WT
lpr/lpr
FasC194V^lpr/lpr
CD3
b
F4/80
c
IgG
d
Complement C3
e
IgG
Corrected Total Cell Fluorescence
25
20
15
10
5
0
*
**
**
WT
lpr/lpr
FasC194V^lpr/lpr
f
C3
Corrected Total Cell Fluorescence
20
15
10
5
0
NS
**
**
WT
lpr/lpr
FasC194V^lpr/lpr

Supplementary Figure 7: Reduced immune phenotype in FasC194V$^{lpr/lpr}$ mouse kidney. Kidney sections from 28-week old age-matched WT, *lpr/lpr*, or FasC194V$^{lpr/lpr}$ mice were stained for either CD3 **(a)** or F4/80 **(b)** by immunohistochemistry, and IgG **(c)** or complement component C3 **(d)** deposition in glomeruli via direct immunofluorescence. Images are representative of minimum 5 mice each genotype for each stain ($n \geq 5$). Quantitation of total cell fluorescence in the glomeruli of each genotype for IgG **(e)** and C3 **(f)**. The mean fluorescence of minimum of 50 glomeruli/mouse were normalized to the average background fluorescence of each image and presented as a corrected total fluorescence. Each data point represents the average data from 50 glomeruli of a single mouse ($n \geq 4$ for each genotype). Mann-Whitney was used for statistical analyses. * $p \leq 0.05$; ** $p \leq 0.01$; NS = Not significant. Scale bar: 200μm.

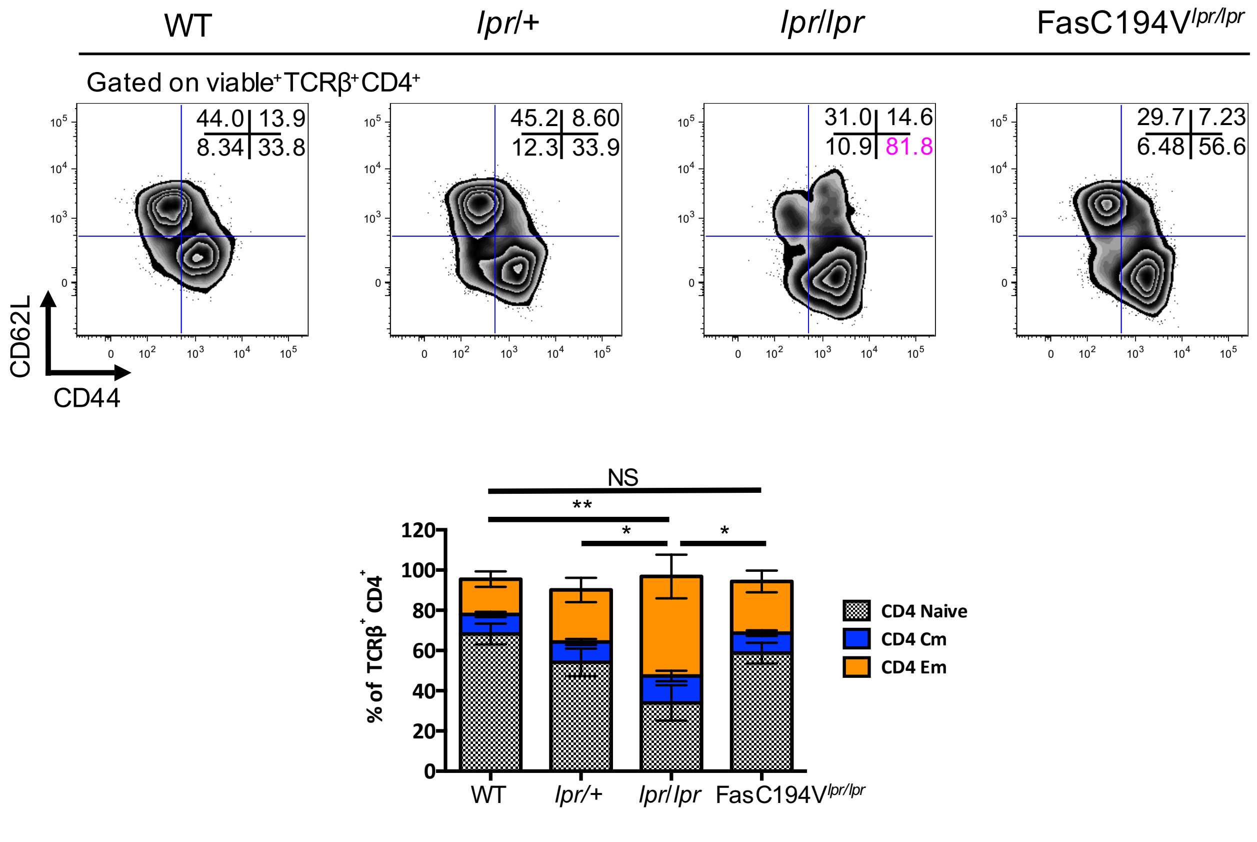

Supplementary Figure 8: Fas C194V prevents effector CD4+ T cell accumulation. Peripheral lymph nodes from 30 week-old WT, *lpr/+*, *lpr/lpr* and FasC194V*lpr/lpr* mice were isolated and stained for surface markers prior to analysis by flow cytometry. Cells were gated TCRβ+CD4+ from the viable population. Similar data was obtained in isolated splenocytes (data not shown). Percentages of CD4+ subsets: Naïve, Central memory (Cm) and Effector memory (Em) are quantitated below. Data are representative of four independent experiments (N=4), with compiled data represented by mean percentage of total +/- SEM. Unpaired t-test was performed to compare Em populations across the genotypes. * $p \leq 0.05$; ** $p \leq 0.01$; NS = Not significant.

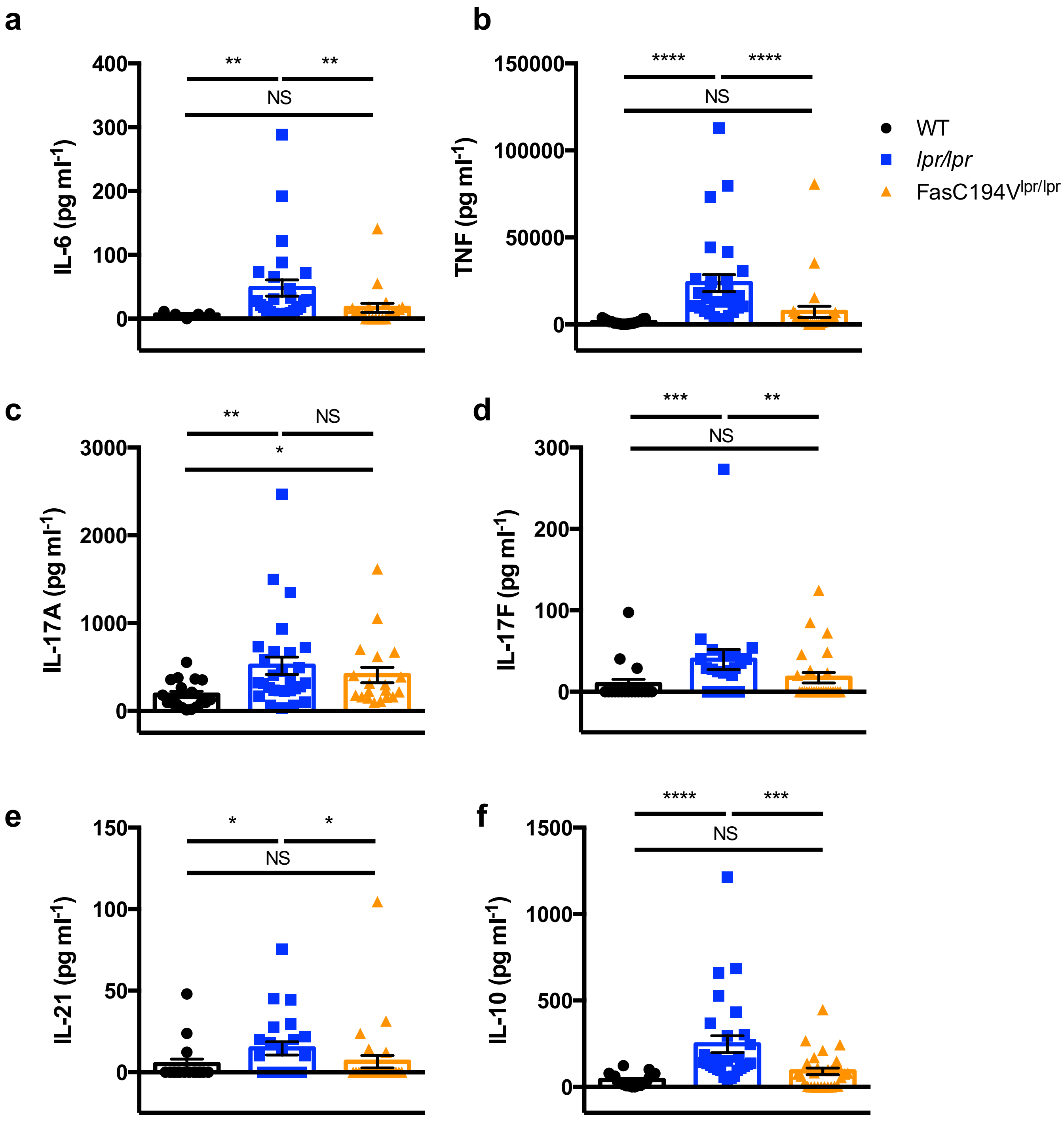

Supplementary Figure 9: Decreased serum inflammatory cytokines in FasC194V$^{lpr/lpr}$ mice. Serum was collected from WT, *lpr/lpr*, or FasC194V^{lprlpr} mice and analyzed for the indicated cytokines via multiplex bead-based protein detection. Minimum of 20 mice per genotype were analyzed (n ≥ 20). Mice were at least 28 weeks of age at the time of serum collection. Mann-Whitney was used for statistical analyses. * $p \leq 0.05$; ** $p \leq 0.01$; *** $p \leq 0.001$; **** $p \leq 0.0001$; NS = Not significant.

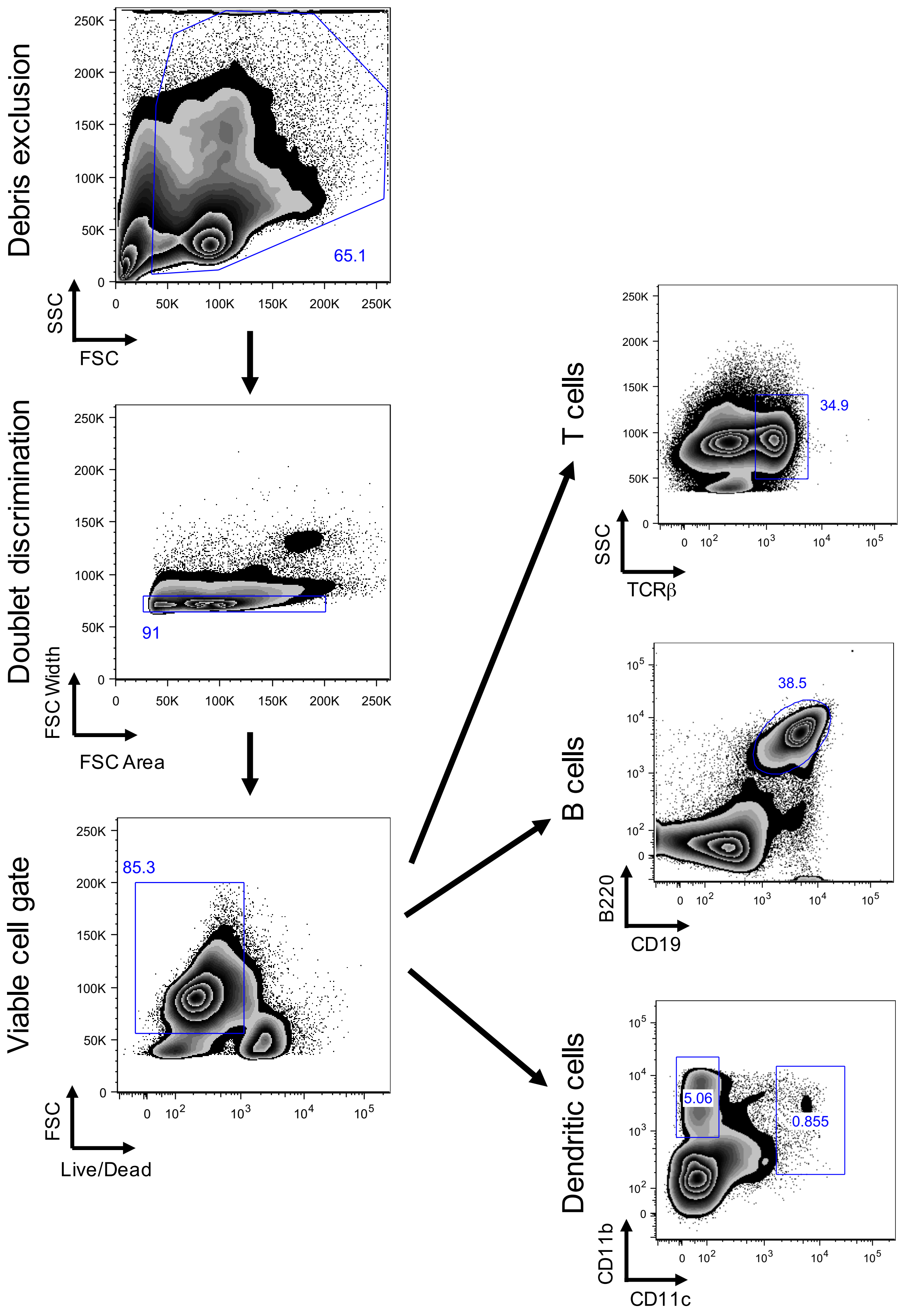

Supplementary Figure 10: Flow cytometry schematic of gating. Example of flow cytometry gating scheme for all data plots involving *ex vivo* mouse cells.

Figure 1d.

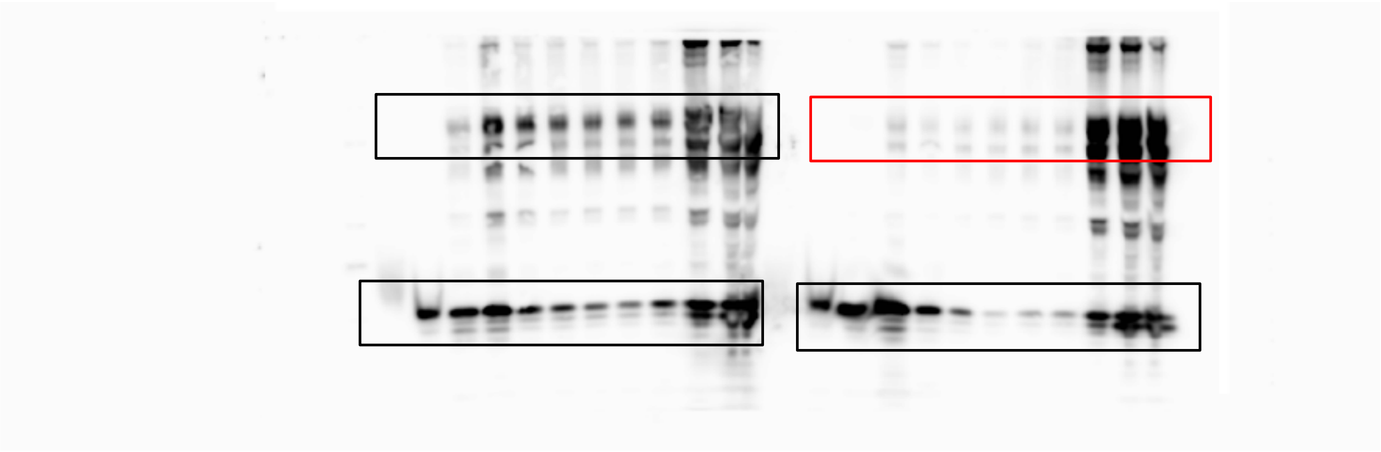

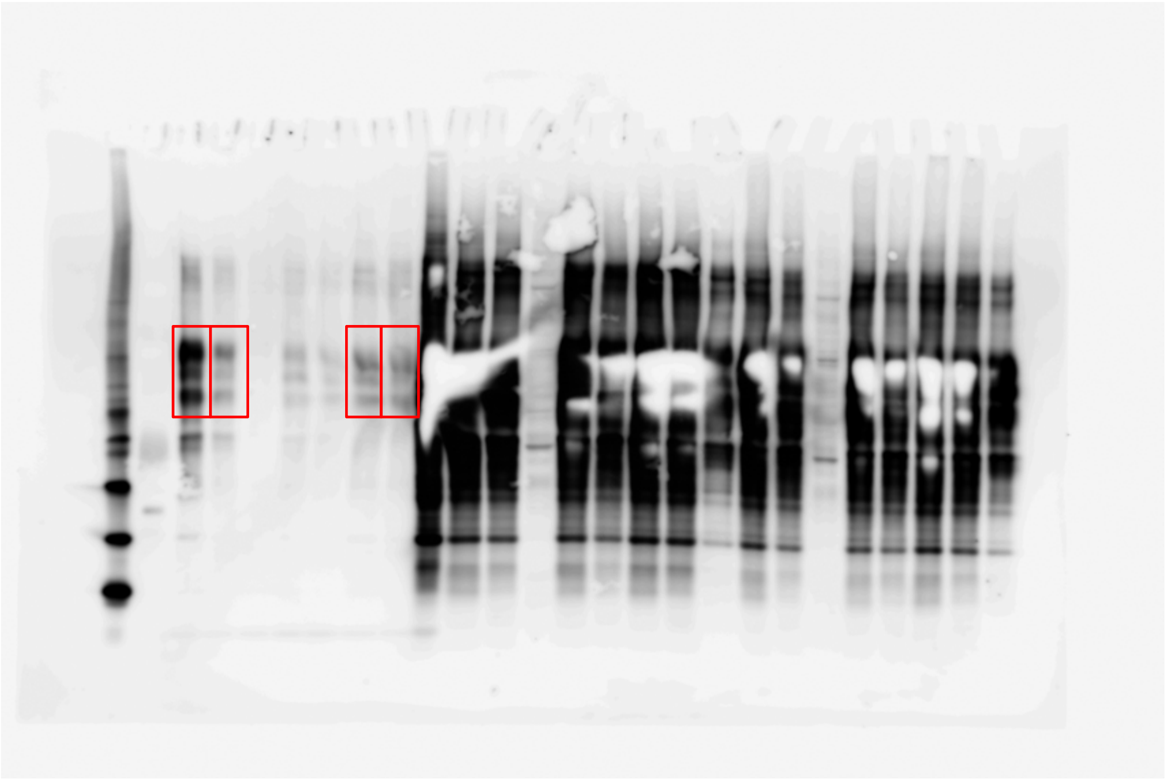

Supplementary Figure 11: Western blot primary data. Uncropped images of primary Western blot data. Red boxes indicate data used in the indicated figures.

Figure 3c.

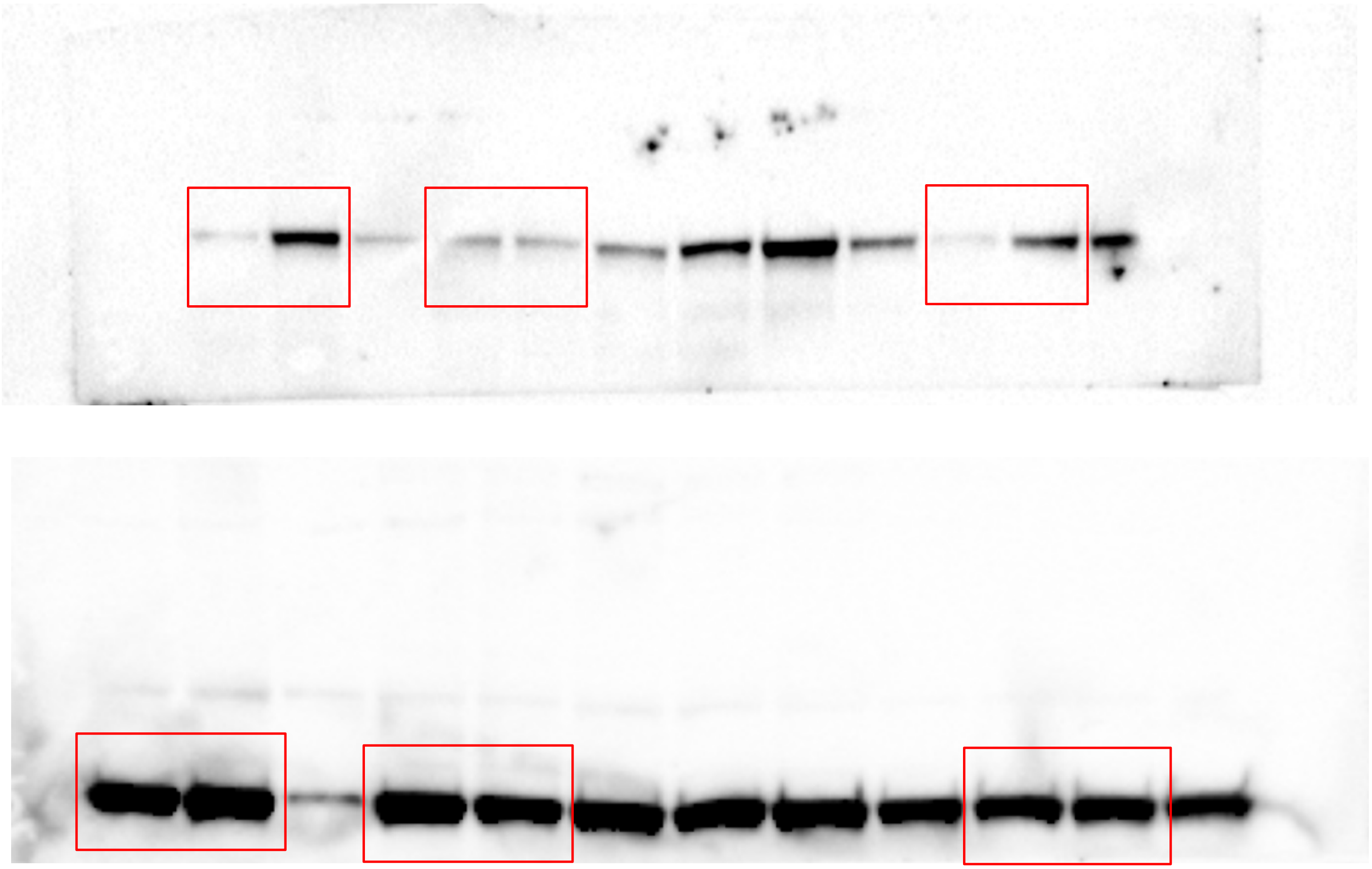

Supplemental Figure 1c.

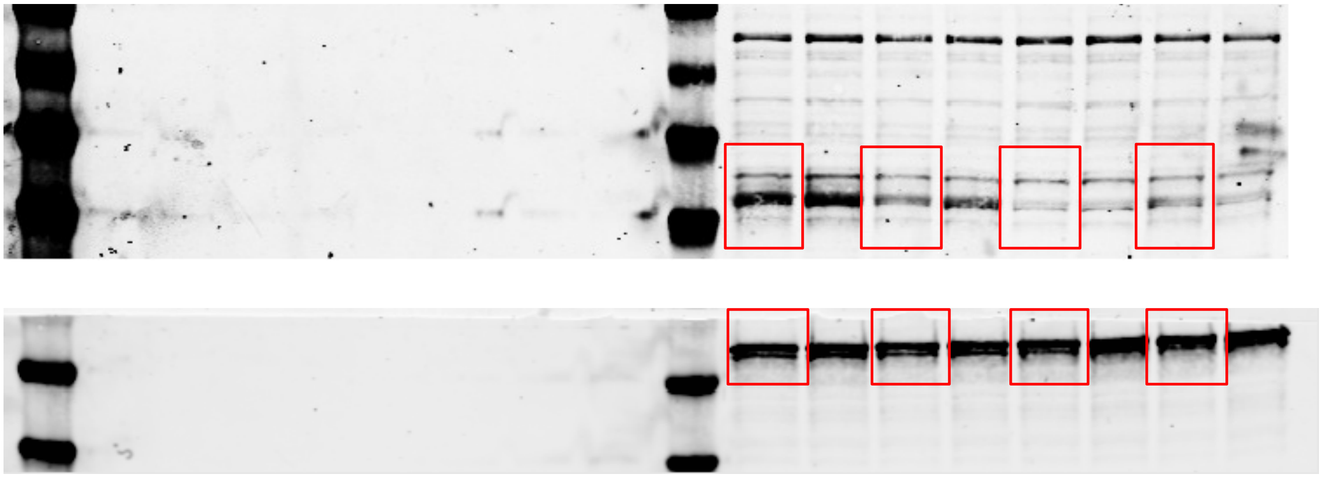

Supplementary Figure 11: Western blot primary data. Uncropped images of primary Western blot data. Red boxes indicate data used in the indicated figures.

Supplemental Figure 2a.

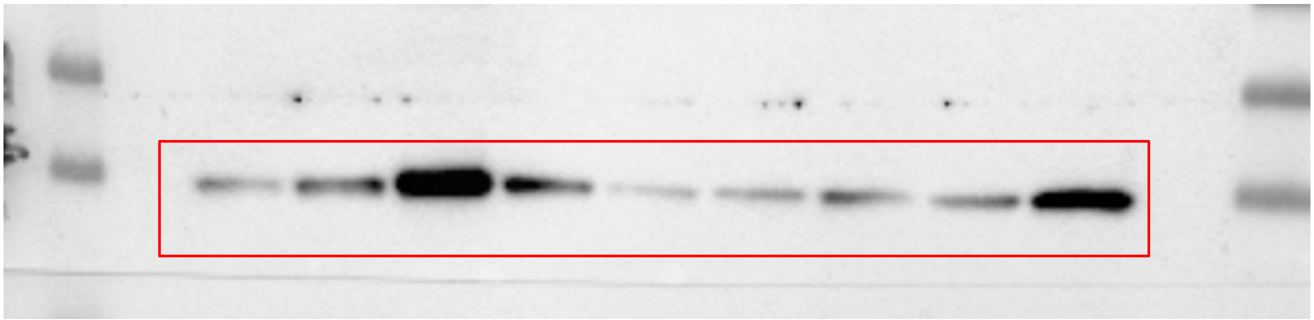

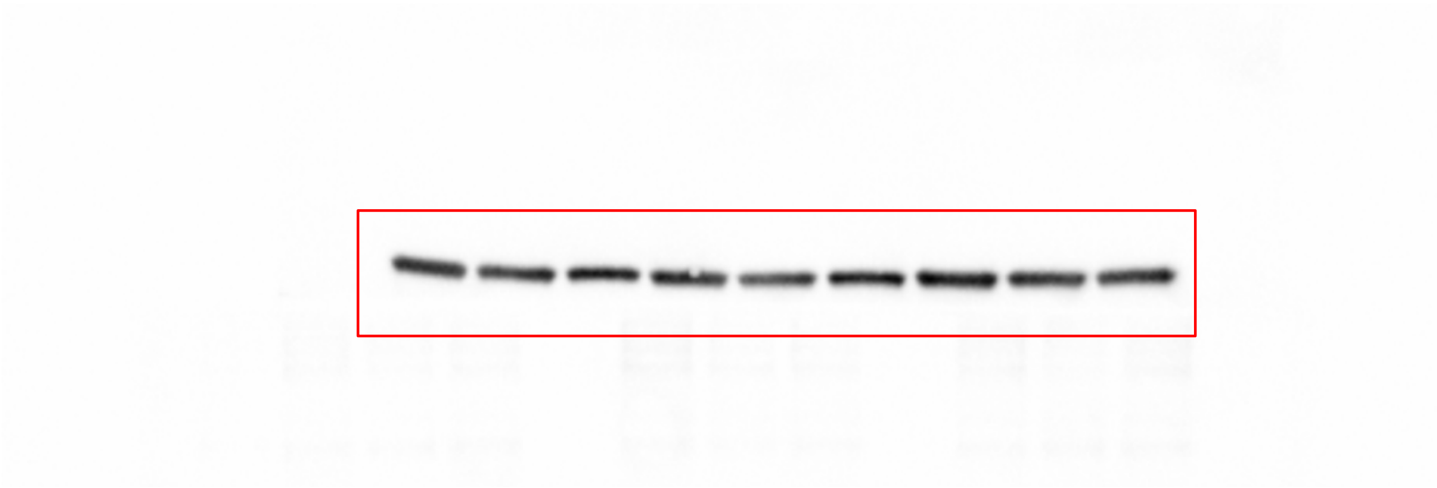

Supplementary Figure 11: Western blot primary data. Uncropped images of primary Western blot data. Red boxes indicate data used in the indicated figures.

Fas mutation	% Fas-induced cell death compared to control
c. 845(+1) g>t	13.8
c. 973A->G ;p. D244G	2.0
c. 970T->C ;p. I243T	3.4
c. 899 del T;p. T219fs	25.2
c. 1106T->A;p. C288X	n.d.
c. 952G->T ;p. G237V	3.7
c. 942C->T ;p. R234X	2.2

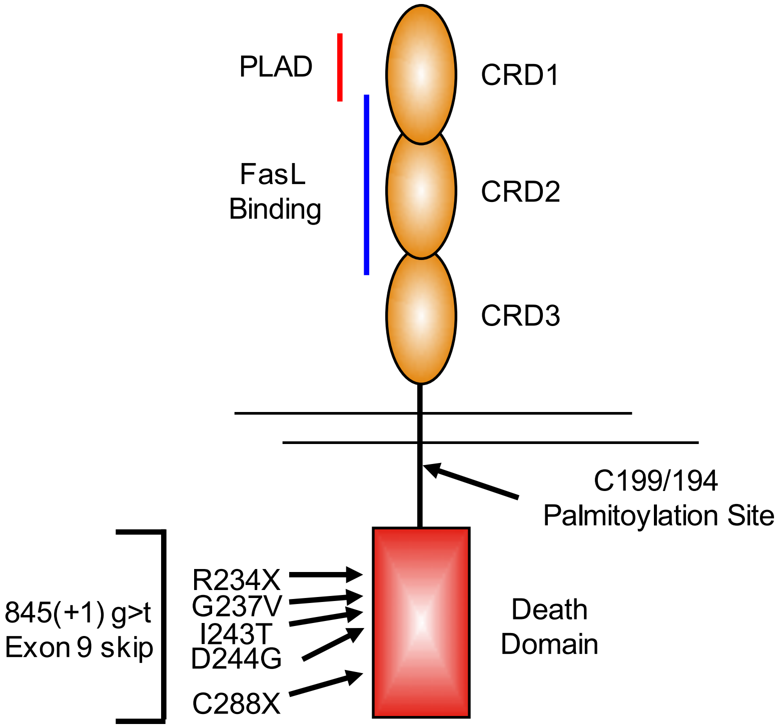

Supplementary Table 1: Listing of Mutations and quantitation of Fas-induced apoptosis in activated T cells from ALPS patients used in Figure 8

	Autoantibody Production	Lymphadenopathy/ Splenomegaly	Increased DC effector B, T cell subsets	'Double Negative' $CD4^{-}CD8^{-}B220^{+}$ T cells	Fas-induced Apoptosis (T, B, DC)	FasL-induced T cell differentiation
Wild-type	-	-	-	-	+	+
lpr/+	-	-	-	-	+	n.d
FasC194V$^{lpr/lpr}$	-	-	-	-	-	+
lpr/lpr	+	+	+	+	-	-

Supplementary Table 2: Summary of the phenotypes of FasC194V$^{lpr/lpr}$ mice compared with the other strains studied
